# Direct and biologically significant interactions of human herpesvirus 8 interferon regulatory factor 1 with STAT3 and Janus kinase TYK2

**DOI:** 10.1371/journal.ppat.1011806

**Published:** 2023-11-20

**Authors:** Zunlin Yang, Qiwang Xiang, John Nicholas

**Affiliations:** Sidney Kimmel Comprehensive Cancer Center at Johns Hopkins, Department of Oncology, Johns Hopkins University School of Medicine, Baltimore, Maryland, United States of America; University of Washington, UNITED STATES

## Abstract

Human herpesvirus 8 (HHV-8) encodes four viral interferon regulatory factors (vIRFs) that target cellular IRFs and/or other innate-immune and stress signaling regulators and suppress the cellular response to viral infection and replication. For vIRF-1, cellular protein targets include IRFs, p53, p53-activating ATM kinase, BH3-only proteins, and antiviral signaling effectors MAVS and STING; vIRF-1 inhibits each, with demonstrated or likely promotion of HHV-8 *de novo* infection and productive replication. Here, we identify direct interactions of vIRF-1 with STAT3 and STAT-activating Janus kinase TYK2 (the latter reported previously by us to be inhibited by vIRF-1) and suppression by vIRF-1 of cytokine-induced STAT3 activation. Suppression of active, phosphorylated STAT3 (pSTAT3) by vIRF-1 was evident in transfected cells and vIRF-1 ablation in lytically-reactivated recombinant-HHV-8-infected cells led to increased levels of pSTAT3. Using a panel of vIRF-1 deletion variants, regions of vIRF-1 required for interactions with STAT3 and TYK2 were identified, which enabled correlation of STAT3 signaling inhibition by vIRF-1 with TYK2 binding, independently of STAT3 interaction. A viral mutant expressing vIRF-1 deletion-variant Δ198–222 refractory for TYK2 interaction and pSTAT3 suppression was severely compromised for productive replication. Conversely, expression of phosphatase-resistant, protractedly-active STAT3 led to impaired HHV-8 replication. Cells infected with HHV-8 mutants expressing STAT3-refractory vIRF-1 deletion variants or depleted of STAT3 displayed reduced vIRF-1 expression, while custom-peptide-promoted STAT3 interaction could effect increased vIRF-1 expression and enhanced virus replication. Taken together, our data identify vIRF-1 targeting and inhibition of TYK2 as a mechanism of STAT3-signaling suppression and critical for HHV-8 productive replication, the importance of specific pSTAT3 levels for replication, positive roles of STAT3 and vIRF-1-STAT3 interaction in vIRF-1 expression, and significant contributions to lytic replication of STAT3 targeting by vIRF-1.

## Author summary

HHV-8 is involved etiologically in Kaposi’s sarcoma, primary effusion lymphoma (PEL), and multicentric Castleman’s disease, which occur predominantly in the context of HIV co-infection. Effective treatments for these diseases, in which both latent and lytic modes of HHV-8 infection are implicated, are lacking. Understanding the molecular biology of HHV-8 and the virus-cellular interactions critical for virus productive and latent infection is important for the development of novel antiviral and therapeutic strategies. HHV-8 vIRF-1 is of demonstrated importance to both latency and lytic replication, contributing to latently-infected PEL cell viability and being required for efficient virus production. This study identifies direct interactions of vIRF-1 with signaling protein STAT3 and STAT-activating TYK2 kinase and correlates the respective interactions with vIRF-1 expression and suppression of STAT3 activation in infected cells along with promotion of productive replication. These data provide a foundation for the development of novel and specific antiviral agents targeting these newly-identified direct interactions.

## Introduction

Human herpesvirus 8 (HHV-8), which is linked causally with the HIV-associated diseases Kaposi’s sarcoma, primary effusion lymphoma (PEL), multicentric Castleman’s disease (MCD) and an MCD-like inflammatory syndrome [1], is the first virus in which viral interferon regulatory factor homologues (vIRFs) were identified [2]. The genes for the four HHV-8 vIRFs have been characterized structurally and all are expressed during lytic (productive) replication, and in the particular context of PEL cells, vIRF-3 is expressed as a *bona fide* latent protein and vIRFs 1 and 2 are expressed to some degree in latency [3–6]. The first functional study of any vIRF demonstrated the ability of vIRF-1 to inhibit interferon-β (IFNβ) signaling and to be oncogenic in *in vitro* and *in vivo* experimental systems [7], revealing its ability to manipulate key cellular antiviral and growth and survival pathways. There have since been numerous reports of interactions of vIRF-1 with cellular proteins, including IRFs 1 and 3, transcriptional coactivators CBP and p300, retinoic acid and interferon-inducible protein GRIM19, p53, the p53 kinase ATM, transcription factors Smad3 and Smad4, antiviral signaling proteins MAVS and STING, and pro-apoptotic BH3-only proteins (BOPs) Bim and Bid [8–10]. Each of these proteins is associated with promotion of interferon signaling and/or apoptosis, and targeting of each by vIRF-1 is inhibitory. Therefore, vIRF-1 is expected to counter these cellular antiviral responses and promote primary infection, latency establishment, and productive replication; indeed, the role of vIRF-1-BOP interactions in virus lytic replication has been demonstrated directly via genetic analyses [11]. It is notable that the other HHV-8 vIRFs also have been demonstrated experimentally to inhibit IFN induction/signaling, examples including vIRF-2 promotion of caspase 3-mediated cleavage of active (phosphorylated) IRF3 and vIRF-3 and -4 association with IRF7, thereby inhibiting functional self-association and heterodimerization with IRF3 [8,9,12–14]. However, despite the many reported interactions and activities of the vIRFs, very few have been examined in the context of infected cells, and the actual functions of the vIRFs and their interactions in virus biology are largely unknown.

Signal transducer and activator of transcription (STAT) factors 1 and 2, in particular, play important roles in the cellular innate immune response to virus infection as mediators of interferon signaling [15,16], and we have reported recently that vIRF-1 inhibits this pathway via direct targeting of STAT1 and STAT2 and inhibition (directly or indirectly) of STAT-kinase TYK2 [17]. STAT3 acts as an inhibitor of such signaling [18] and also functions to promote cell viability and proliferation; STAT3 has been implicated in many types of human cancer, where it is found at elevated active levels, caused by induced signaling (e.g., via cytokine dysregulation) and/or activating mutations that promote the phosphorylated form of STAT3 [19–23]. With respect to HHV-8, STAT3 is activated by *de novo* infection of endothelial cells and in latently infected endothelial and PEL cells and plays critical roles in sustaining the viability of latently infected cells and enabling efficient productive replication [24–27]. STAT3-dependent expression of survivin is a key mechanism sustaining the viability of latently infected PEL cells [25]. The mechanism of STAT3 promotion of productive replication in these and other cell types has not been established, but signaling by the virus-encoded IL-6 homologue (vIL-6) through the gp130 cytokine receptor is a major mechanism of STAT3 activation that contributes to productive replication [27,28]. While STAT3 signaling is of demonstrable importance to HHV-8 latent and lytic biology, it seems reasonable to hypothesize that there are mechanisms by which this signaling can be attenuated and fine-tuned to achieve optimal conditions for both phases of infection. Furthermore, active-STAT3 levels appear to regulate the maintenance of latency versus the switch to lytic replication in the context of PEL cells, at least, a mechanism involving the STAT3-induced expression of the transcriptional corepressor KAP1/TRIM28, which represses the HHV-8 immediate-early transcriptional activator and lytic inducer RTA [29].

Here, we identify novel properties of vIRF-1, namely the direct physical targeting of STAT3 and one of its Janus kinases, TYK2, and the inhibition of STAT3 activation via the latter interaction. Using genetic approaches, we also identify the biological significance of vIRF-1 and specifically vIRF-1-TYK2 interaction with respect to suppression of STAT3 activation and signaling in infected cells and promotion of productive replication. While vIRF-1-STAT3 interaction appears not to regulate levels of active STAT3, we present evidence that the interaction is important for virus production and can promote vIRF-1 expression. Our data reveal the first example of HHV-8 vIRF regulation of STAT3 activity and the importance of such regulation for optimal lytic replication and identify STAT3 as a positive factor for expression of vIRF-1, a pro-replication factor, revealing mutual and biologically significant regulation of these proteins.

## Results

### Interaction of vIRF-1 with STAT3

**Fig 1:**
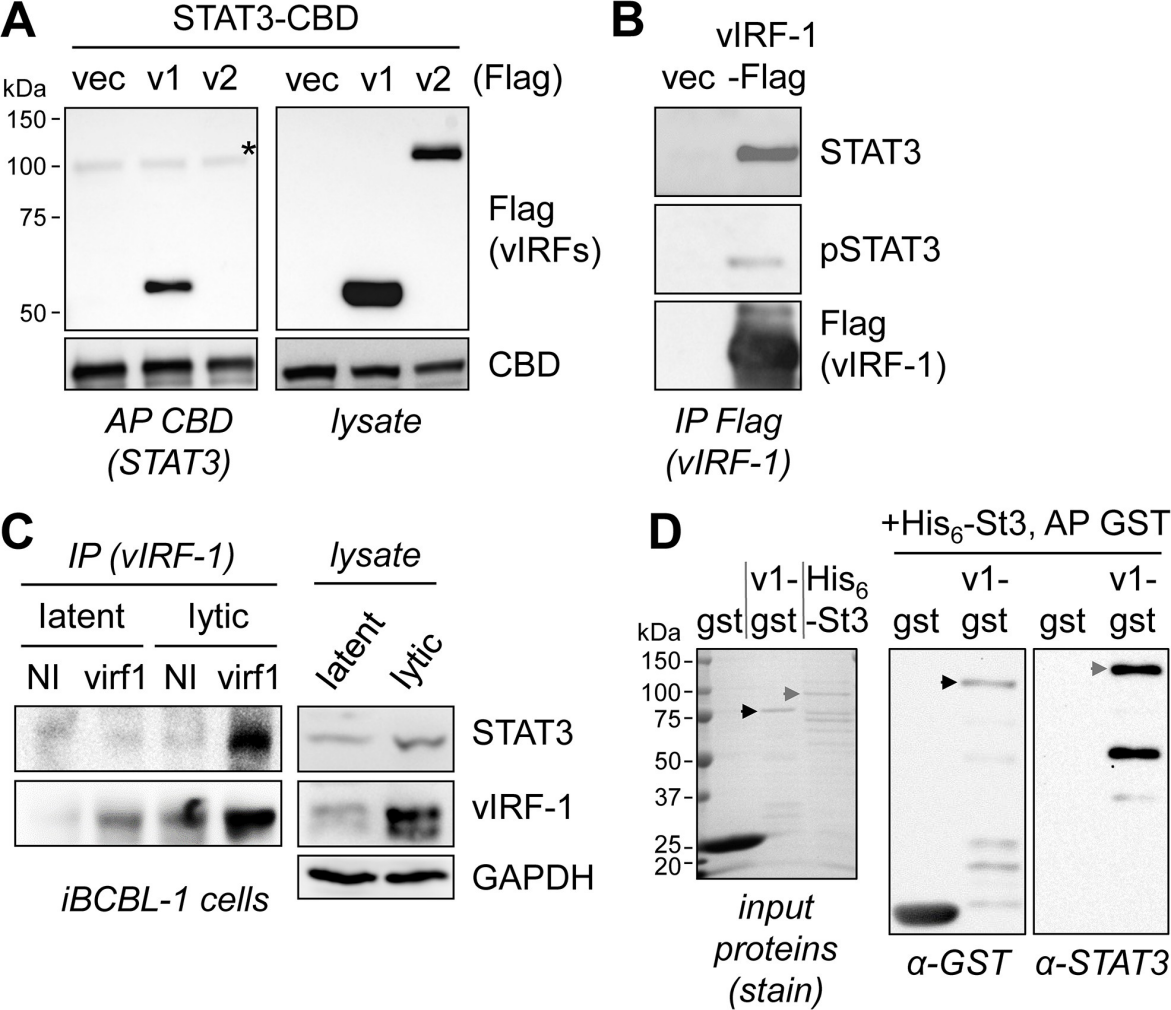
Interaction of vIRF-1 with STAT3. (A) Transfection-based coprecipitation assays were carried out using transfectant lysates containing CBD-tagged STAT3 and Flag-tagged vIRF-1, or vIRF-2 (for comparison), or derived from 293T cells transfected with STAT3-CBD and empty vector (vec) plasmids. Affinity precipitation (AP) of STAT3-CBD utilized chitin-conjugated beads, and precipitates and lysates were analyzed by immunoblotting for detection of coprecipitated vIRF and input proteins. A non-specific band in the AP/Flag blot is indicated (*). (B) Reciprocal coprecipitation experiment in which immunoprecipitation (IP) of vIRF-1-Flag was carried out, followed by immunoblotting to detect tyrosine-phosphorylated STAT3 (pSTAT3) and total STAT3. (C) Immunoprecipitation (IP) of vIRF-1 from lysates of latent and 2-day lytic (Dox-treated) TRExBCBL1-RTA (iBCBL-1) PEL cells and immunoblot detection of coprecipitated STAT3. Rabbit vIRF-1 antiserum (virf1) or non-immune rabbit antiserum (NI, negative control) were used for IP. (D) AP-based analysis of direct vIRF-1-STAT3 interaction using bacterially-derived and purified GST-tagged vIRF-1 (v1-gst), or GST alone (gst), and His_6_-tagged STAT3 (His_6_-St3). The black and grey arrowheads indicate full-length vIRF-1-GST and His_6_-STAT3 bands, respectively. Input proteins were visualized on a Coomassie-stained gel (left) to assess purity and integrity of the proteins; there was evidence of some degradation of each protein on this and the respective immunoblots (α-GST, α-STAT3).

Mass spectrometry analysis of proteins coprecipitating with affinity-tagged vIRF-1 identified STAT3, along with several other cellular proteins including USP7 [30], as a potential interaction partner of vIRF-1. To verify this, we undertook transfection-based coprecipitation assays using either STAT3-CBD (chitin-binding domain) affinity precipitation (AP) or Flag-based immunoprecipitation (IP) of tagged vIRF-1. STAT3-CBD could precipitate vIRF-1, but not vIRF-2 (included for comparison), from cotransfected cell lysates (Fig 1A); the reciprocal precipitation, of vIRF-1-Flag, identified coprecipitation of endogenous STAT3, including active, tyrosine-phosphorylated STAT3 (pSTAT3) (Fig 1B). Immunoprecipitation of virus-expressed vIRF-1 from lysates of latent and lytically reactivated TRExBCBL1-RTA (“iBCBL-1”) PEL cells [31] also identified coprecipitated STAT3, above background levels detected using control antiserum (with which some non-specific precipitation of vIRF-1 was evident) (Fig 1C). To determine if vIRF-1 interaction with STAT3 was direct, we isolated GST-fused vIRF-1, or GST (control), and His_6_-tagged STAT3 from respective vector-transformed bacteria for use in *in vitro* binding assays. STAT3 coprecipitated with vIRF-1-GST but not GST alone (Fig 1D), demonstrating direct, bimolecular interaction of (unphosphorylated) STAT3 and vIRF-1.

### STAT3 regulation by vIRF-1

**Fig 2:**
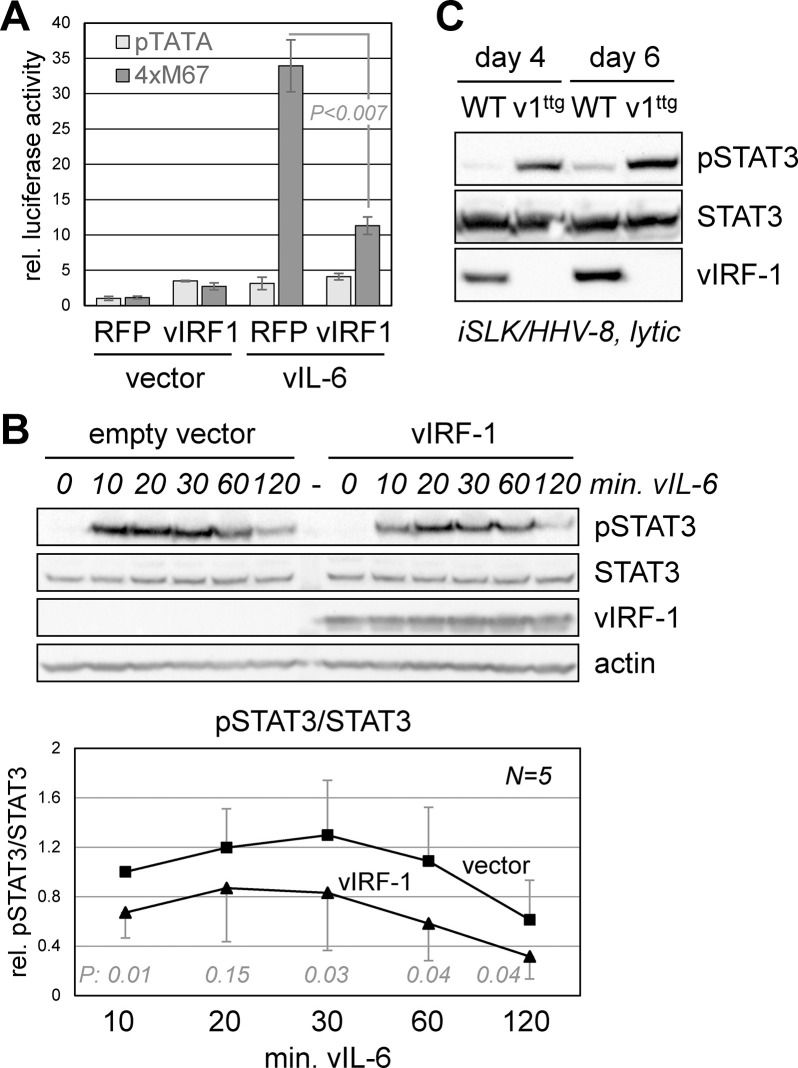
Influence of vIRF-1 on STAT3 activation and function. (A) Assessment of active STAT3 using a STAT3-responsive luciferase reporter (4xM67), or TATA-only basal parental plasmid (pTATA), in 293T cells cotransfected with RFP (negative control) or vIRF-1 expression vector, either lacking or coexpressing STAT3-activating vIL-6. Average values from triplicate transfections are shown along with standard deviations; student t-test (two-tailed) *P* value is shown for vIRF-1-suppressed reporter activity in vIL-6-expressing cells. (B) 293T cultures transfected with empty vector or vIRF-1 expression plasmid were stimulated with vIL-6-conditioned medium for different times and levels of active, tyrosine-phosphorylated STAT3 (pSTAT3) were assessed in derived whole-cell lysates by immunoblotting. β-actin and STAT3 (loading controls) and vIRF-1 (to confirm appropriate expression) were also detected. The chart shows levels of pSTAT3, normalized to total-STAT3, relative to pSTAT3 in empty-vector-transfected cells at 10 min. of vIL-6 stimulation from this and four additional experiments; standard deviations from average values and student t-test *P* values are shown for each timepoint. (C) Immunoblotting was carried out to assess levels of pSTAT3 in iSLK cells infected with either wild-type (WT) or vIRF-1-knockout (v1^ttg^) HHV-8 and lytically-reactivated by Dox/NaB-treatment for either 4 or 6 days.

We next investigated if vIRF-1 could regulate STAT3-mediated signaling. Initially, we utilized a STAT3-responsive reporter, 4xM67 pTATA TK-luc [32], here referred to as 4xM67-Luc, or control vector containing only the TATA box; these were transfected into 293T cells along with a plasmid encoding STAT3-activating viral interleukin-6 (vIL-6), or empty vector control, and either vIRF-1 expression plasmid or red fluorescent protein (RFP)-encoding vector (negative control). Analysis of the respective cell lysates showed that luciferase was induced by vIL-6 and that this activated level was decreased in the presence of coexpressed vIRF-1 (Fig 2A). The TATA-only promoter-luciferase control showed little response to vIL-6, consistent with specific effects via STAT3 binding to the M67 *cis*-elements. To determine if vIRF-1 function was related to inhibition of STAT3 activation, rather than effects on its activity after activation, we analyzed transfected cell extracts for relative levels of STAT3 tyrosine-705 (Y_705_) phosphorylation in response to acute stimulation by vIL-6 in the absence or presence of vIRF-1. Cells were harvested at various times (10 to 120 minutes) after addition of vIL-6-containing conditioned medium. Immunoblotting of cell extracts identified vIRF-1 suppression of phospho-STAT3 (pSTAT3) (Fig 2B); these and replicate data were quantified and analyzed statistically (Fig 2B chart). Notably, there was no detectable change in the duration of induced pSTAT3, indicating that inactivation via pSTAT3 dephosphorylation was unaffected by vIRF-1.

To assess STAT3 regulation by vIRF-1 in the context of infected cells, we took advantage of a vIRF-1-knockout virus (BAC16.vIRF-1^ttg^) that we generated previously [30], in which the initiator-ATG codon of the vIRF-1 open reading frame was changed to TTG. Epithelial iSLK cells [33], inducible by doxycycline (Dox) treatment for the expression of the viral immediate-early transactivator RTA, were infected with equivalent infectious doses of either wild-type or vIRF-1-knockout virus, and the latently infected cells were induced into lytic replication by Dox and sodium butyrate (NaB) treatment, for 4 or 6 days. Immunoblotting of cell extracts identified substantially increased pSTAT3 levels in vIRF-1-null (v1^ttg^) relative to wild-type (WT) virus-infected cells (Fig 2C). These data demonstrate that vIRF-1 can suppress active (Y_705_-phosphorylated) STAT3 in lytically-infected cells, as it can in transfected cells.

### STAT3 targeting in the suppression of pSTAT3 by vIRF-1

**Fig 3:**
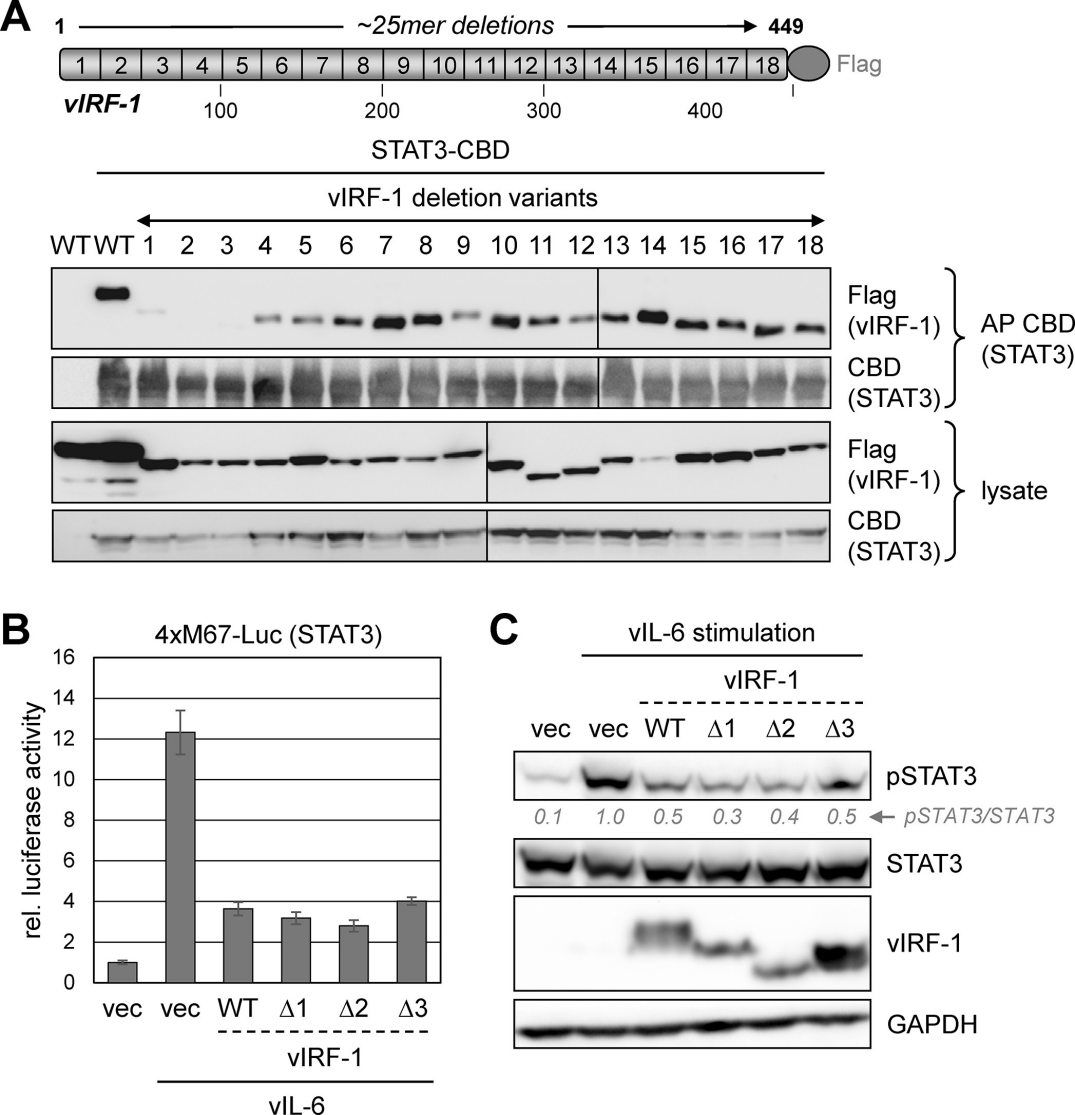
Dissociation of vIRF-1 targeting and functional suppression of STAT3. (A) Mapping of vIRF-1 regions required for interaction with STAT3. A panel of vectors expressing Flag-tagged vIRF-1 variants comprising scanning ~25-residue deletions was used along with STAT3-CBD expression plasmid to transfect 293T cultures, and derived cell lysates were analyzed by chitin-based affinity-precipitation (AP) and Flag-immunoblotting for vIRF-1-STAT3 interaction. Solid lines denote separate immunoblot membranes. (B) Deletion-variants 1, 2 and 3 (Δ1, Δ2, Δ3) were compared with full-length, wild-type (WT) vIRF-1 in a STAT3-responsive luciferase reporter (4xM67-Luc) assay for suppression of reporter induction by coexpressed vIL-6 in transfected 293T cells. Triplicate transfectants were analyzed; data shown represent mean values relative to uninduced activity (without vIL-6, set at 1), and error bars represent standard deviations from the means. (C) A similar experiment was carried out using immunoblotting for pSTAT3 to detect directly relative levels of vIL-6-activated STAT3 in response to wild-type and deletion-variant vIRF-1 expression. Conditioned medium containing vIL-6 was applied for 10 min. to stimulate STAT3 activation.

To investigate the significance of vIRF-1-STAT3 interaction for the inhibition of STAT3 activation by vIRF-1, we sought first to identify vIRF-1 variants defective for the interaction and then test these for activity. A series of ~25mer deletion variants of vIRF-1 were used in STAT3-CBD-based coprecipitation assays; each of the first three deletions (Δ1-Δ3), covering vIRF-1 residues 1 to 72, led to impaired (Δ1) or loss of STAT3 binding (Fig 3A). However, the mutations did not affect recently reported [17] interactions of vIRF-1 with STAT1 and STAT2 (S1 Fig). Importantly, each of the variants was found to be fully active with respect to suppression vIL-6-induced STAT3 signaling, as determined by reporter assay (Fig 3B), revealing that this activity occurs independently of vIRF-1 interaction with STAT3. Immunoblot measurements of vIL-6-induced pSTAT3 in the presence of native vIRF-1 or the N-terminal deletion variants verified activities of the latter in pSTAT3 suppression (Fig 3C).

### Physical and functional interactions of vIRF-1 with TYK2 Janus kinase

**Fig 4:**
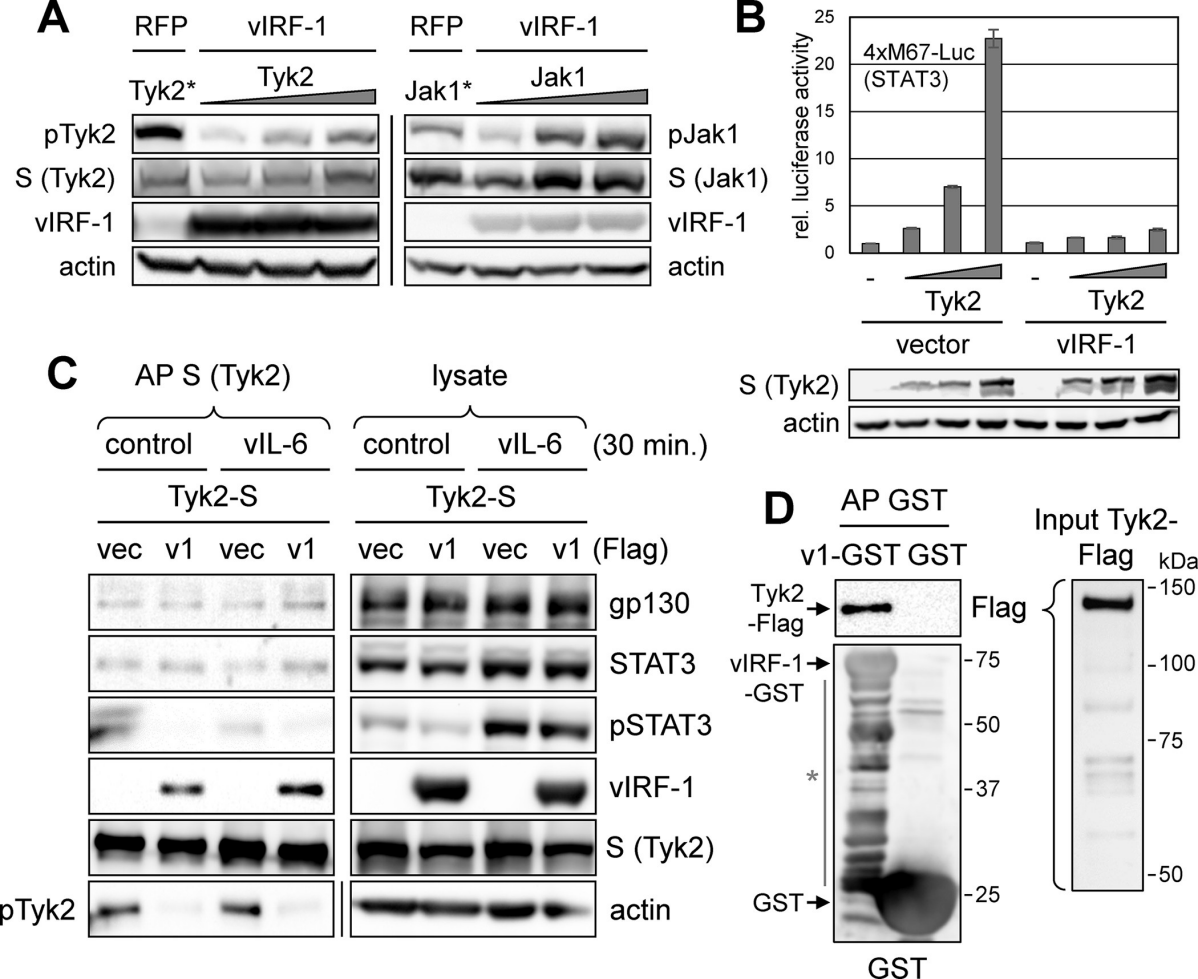
Functional and physical interactions of vIRF-1 with TYK2. (A) Immunoblot assessment of levels of active, autophosphorylated pTYK2 and pJAK1, reflecting activities of the respective Janus kinases, as a function of vIRF-1 expression. Cultures of 293T cells were transfected with different amounts of the kinase vectors (0.15–0.60 μg) along with vIRF-1 expression plasmid; one dose (*, 0.5 μg) of TYK2 or JAK1 vector was transfected with RFP-expressing plasmid (negative control). Relative levels of TYK2, JAK1, and vIRF-1 expression along with β-actin (loading control) were assessed by immunoblotting. (B) Luciferase reporter (4xM67-Luc)-based analysis of functional suppression of TYK2 activity by vIRF-1. 293T cells were cotransfected with the STAT3-responsive reporter and different amounts of TYK2 expression plasmid, along with either empty vector (vector) or vIRF-1 expression plasmid. Luciferase activities in cell extracts are shown relative to levels (set at 1) for empty-vector-transfected cells lacking TYK2 overexpression (-). Average values from triplicate samples along with standard deviations are shown. The blots below the chart verify appropriate, vector-dose-dependent expression of TYK2 (in combined triplicate lysates). (C) Analysis of potential vIRF-1 effects on TYK2-STAT3 and TYK2-gp130 interactions in response to vIL-6 stimulation. Transfected-cell lysates containing TYK2-S in the absence (vec) or presence of vIRF-1 (v1) were subjected to S-protein-based AP and precipitated material was immunoblotted for detection of coprecipitated gp130 and total and phosphorylated STAT3. Tyrosine-phosphorylated (active) TYK2 (pTyk2) was also detected, along with vIRF-1. (D) Interaction of bacterially-produced and purified GST-tagged vIRF-1 (v1-GST) and Flag-tagged TYK2. AP of GST-vIRF-1 or GST (control) with glutathione beads and Flag-immunoblotting for coprecipitated TYK2 identified specific and direct interaction of vIRF-1 and TYK2. The asterisk indicates degradation products.

Having dissociated vIRF-1 targeting of STAT3 from suppression of its activation, we next investigated the effects of vIRF-1 on STAT3-activating Janus kinases. We recently reported intracellular binding of TYK2 and JAK1 by vIRF-1 and inhibition of TYK2 autophosphorylation by vIRF-1 [17]. To confirm specific inhibition of TYK2 by vIRF-1, we overexpressed TYK2 and JAK1 Janus kinases in transfected 293T cells cotransfected with either vIRF-1 or RFP (control) expression plasmid. Autophosphorylation of each kinase was induced in a dose-dependent manner, as detected by pTYK2 and pJAK1 immunoblotting, but only TYK2 phosphorylation was suppressed by vIRF-1 (Fig 4A). Inhibition of TYK2 activity by vIRF-1 was evident also in a reporter assay using STAT3-responsive 4xM67-Luc reporter plasmid (Fig 4B). Reflecting vIRF-1-reduced phosphorylation of TYK2 and STAT3 activation, we found that pSTAT3, but not total STAT3, association with affinity-precipitated TYK2-S was reduced significantly in the presence of vIRF-1, with or without vIL-6 stimulation (Fig 4C). Probing for gp130 in TYK2-S affinity-precipitates revealed no detectable effect of vIRF-1 on gp130-TYK2 association, ruling this out as a potential means of pSTAT3 suppression in vIL-6-stimulated cells. Finally, to determine if vIRF-1 can interact directly with TYK2, bacterially-derived and affinity-purified proteins were utilized for affinity precipitation; Flag-tagged TYK2 was co-precipitated with GST-tagged vIRF-1 but not by GST (negative control) (Fig 4D). Taken together, our data indicate that vIRF-1 suppression of pSTAT3 is mediated by direct inhibition of TYK2 by vIRF-1.

### TYK2 contribution to STAT3 activation in HHV-8-infected cells

**Fig 5:**
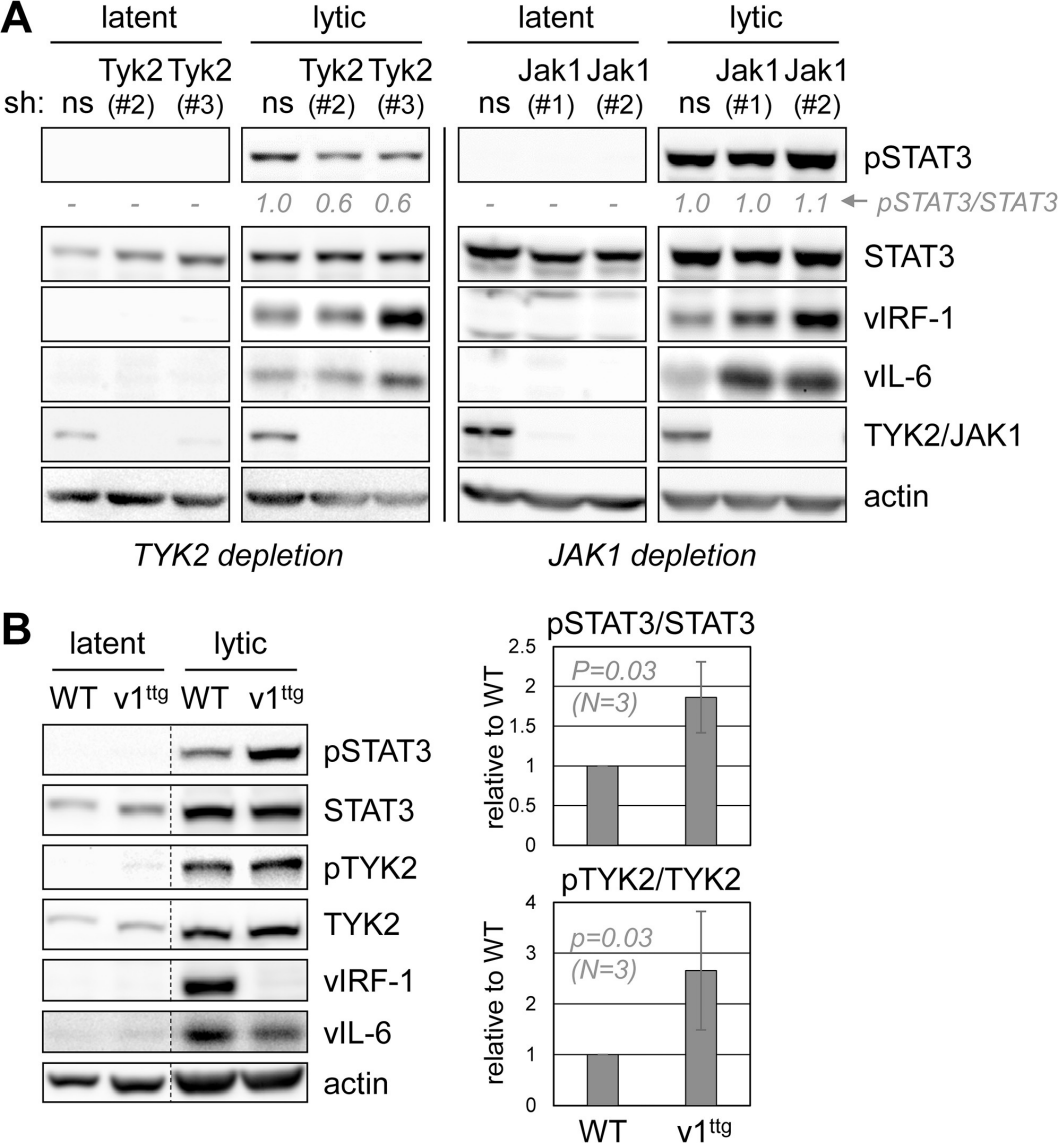
JAK and vIRF-1 regulation of pSTAT3 in HHV-8-infected cells. (A) HHV-8 (BAC16) infected iSLK cells were depleted of either TYK2 or JAK1 via lentiviral vector-mediated shRNA expression, using two different shRNAs for each target; non-silencing (ns) shRNA was used as a control. The cultures were left untreated (latent) or treated with Dox/NaB to induce lytic replication, and cells were harvested after six days for immunoblot analysis of pSTAT3, total-STAT3, and lytically expressed vIRF-1 and vIL-6. Relative levels of pSTAT3, normalized to total-STAT3, are shown below the pSTAT3 blots. (B) Analysis of pSTAT3 and pTYK2 levels as a function of vIRF-1 expression in iSLK cells, infected with native (WT) and vIRF-1-knockout (v1^ttg^) BAC16 viruses. Cultures were ether untreated (latent) or treated with Dox/NaB (lytic) for six days. Immunoblotting of whole-cell lysates was carried out to detect levels of pSTAT3 and pTYK2 relative to total STAT3 and TYK2 and lytic markers vIRF-1 and vIL-6. Charts show quantified data from triplicate lytic cultures, with WT values set at 1. Student’s t-test *P* values (two-tailed) are shown.

Preceding data identified involvement of vIRF-1 in pSTAT3 suppression in lytically infected iSLK cells (Fig 2C). In this context, we used depletion to test the contribution of TYK2, and also JAK1, to lytic cycle-activated STAT3. In both latently and lytically infected cells, TYK2 and JAK1 depletions via lentiviral vector-delivered shRNAs were highly effective (Fig 5A). Active, pSTAT3 was detected only in lytically infected cultures, and depletion of TYK2, but not JAK1, led to reductions in pSTAT3 levels. These data demonstrate the involvement of TYK2 in pSTAT3 activation in lytically infected iSLK cells. Interestingly, JAK1 depletion and shRNA#3-mediated TYK2 depletion led to increased expression of lytic markers vIRF-1 and vIL-6, but the underlying mechanism is unknown and will require further, in-depth investigation. A similar experiment was performed to monitor activating phosphorylation of both STAT3 and TYK2 in response to vIRF-1 ablation (in HHV-8 recombinant BAC16.vIRF-1^ttg^) in lytically infected iSLK cells. Again, pSTAT3 was not readily detected in untreated, latently infected cells, but was induced following lytic induction. Levels of pSTAT3 were higher in BAC16.vIRF-1^ttg^-infected cells relative to wild-type virus-infected cells, consistent with data of Fig 2C, and pTYK2 levels were similarly increased in response to lytic induction and vIRF-1 ablation (Fig 5B). These data indicate that vIRF-1 can regulate pSTAT3 via TYK2 inhibition in the context of infection.

### Disruption and functional analyses of vIRF-1-TYK2 interaction

**Fig 6:**
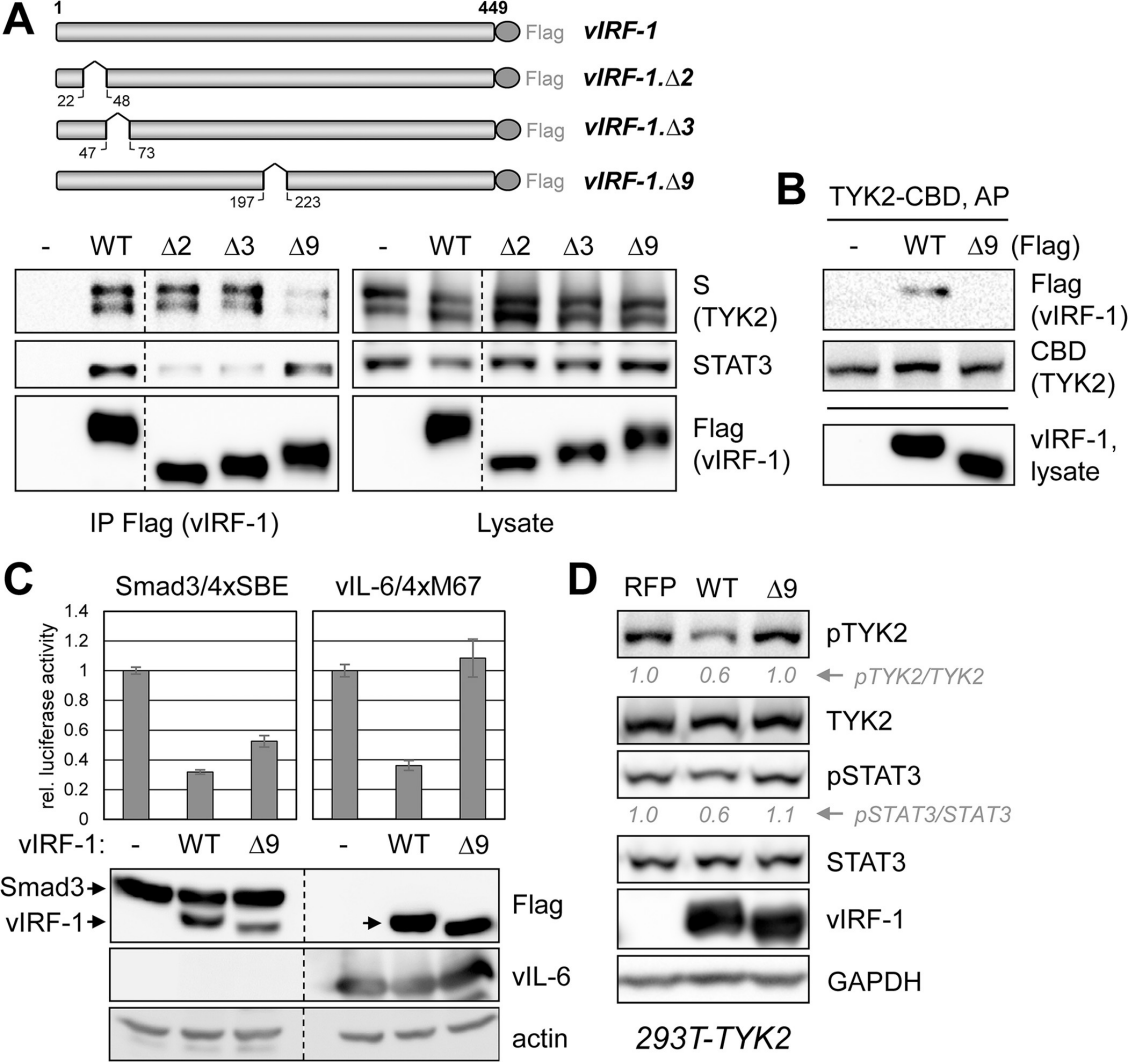
Mapping and functional analysis of vIRF-1 interaction with TYK2. (A) Verification of screening-identified vIRF-1Δ9 (deleted of residues 198–222) as defective for TYK2 interaction. This variant, along with STAT3-binding-defective Δ2 and Δ3 (Fig 3A), were compared for TYK2 and STAT3 interactions by immunoprecipitation (IP) assay employing vIRF-1-Flag as “bait” and immunoblotting for vector-expressed S-tagged TYK2 or endogenous STAT3 in the precipitates. (B) Reciprocal precipitation for vIRF-1-TYK2 interaction, using affinity precipitation (AP) of TYK2-CBD and detection of coexpressed vIRF-1 or vIRF-1Δ9. (C) Testing of vIRF-1Δ9 for suppression of vIL-6-induced STAT3 signaling by transfection-based 4xM67-Luc reporter assay. The variant was inactive in this assay but retained inhibitory effect on the Smad-responsive 4xSBE-Luc reporter, used in parallel transfections. Immunoblotting for Flag-tagged vIRF-1, Smad3, and vIL-6 confirmed appropriate expression of the respective proteins in transfected-cell lysates (combined triplicates). (D) Diminished functional interaction of vIRF-1Δ9 with TYK2 was confirmed by analyzing cell lysates for pTYK2 and pSTAT3 levels in lentiviral vector-transduced, TYK2-overexpressing 292T cells (293T-TYK2) transfected with vIRF-1, vIRF-1Δ9, or RFP (negative control) expression plasmids. Levels of pTYK2 and pSTAT3, normalized to TYK2 and STAT3, respectively, and expressed relative to levels (set at 1) in the RFP vector-transfected culture are shown below the respective blots.

Preliminary coprecipitation-based screening of TYK2 binding by our panel of vIRF-1 deletion variants (Fig 3A) identified vIRF-1Δ9 (Δ198–222) as defective for TYK2 interaction. A follow-up coprecipitation experiment compared this variant with wild-type vIRF-1 and STAT3-refractory variants Δ2 and Δ3 (Fig 3) for interactions with TYK2 and STAT3. The vIRF-1Δ9 variant was confirmed to be deficient for interaction with TYK2, but not STAT3, whereas the reverse was true of vIRF-1 variants Δ2 and Δ3 (Fig 6A). TYK2-based affinity precipitations also verified that the Δ9 variant of vIRF-1 was defective for TYK2 interaction (Fig 6B).

The vIRF-1Δ9 variant was further tested in reporter-based functional assays for expected loss of STAT3-signaling suppression and also, to test specificity, its maintenance of previously identified Smad transcription factor suppression by vIRF-1 [34]. Reporter plasmids containing luciferase-linked *cis*-promoter elements 4xM67 (STAT3) and 4xSBE (Smad) were transfected into 293T cells along with empty, vIRF-1 or vIRF-1Δ9 expression vectors. STAT3 suppression was lost as a result of the Δ9 mutation, while this variant and wild-type vIRF-1 were able to inhibit Smad-reporter expression (Fig 6C). In lenti-TYK2 vector-transduced 293T (293T-TYK2) cells expressing vIRF-1, vIRF-1Δ9, or RFP (control), we found that vIRF-1Δ9 was defective for suppression of TYK2 overexpression-induced pTYK2 and pSTAT3, as determined by immunoblotting (Fig 6D). Together, these data indicate that vIRF-1 interaction with TYK2 is necessary for its inhibition, via interference with activating phosphorylation, and suppression of downstream signaling.

### STAT3 signaling as a function of vIRF-1-TYK2 disruption in infected cells

**Fig 7:**
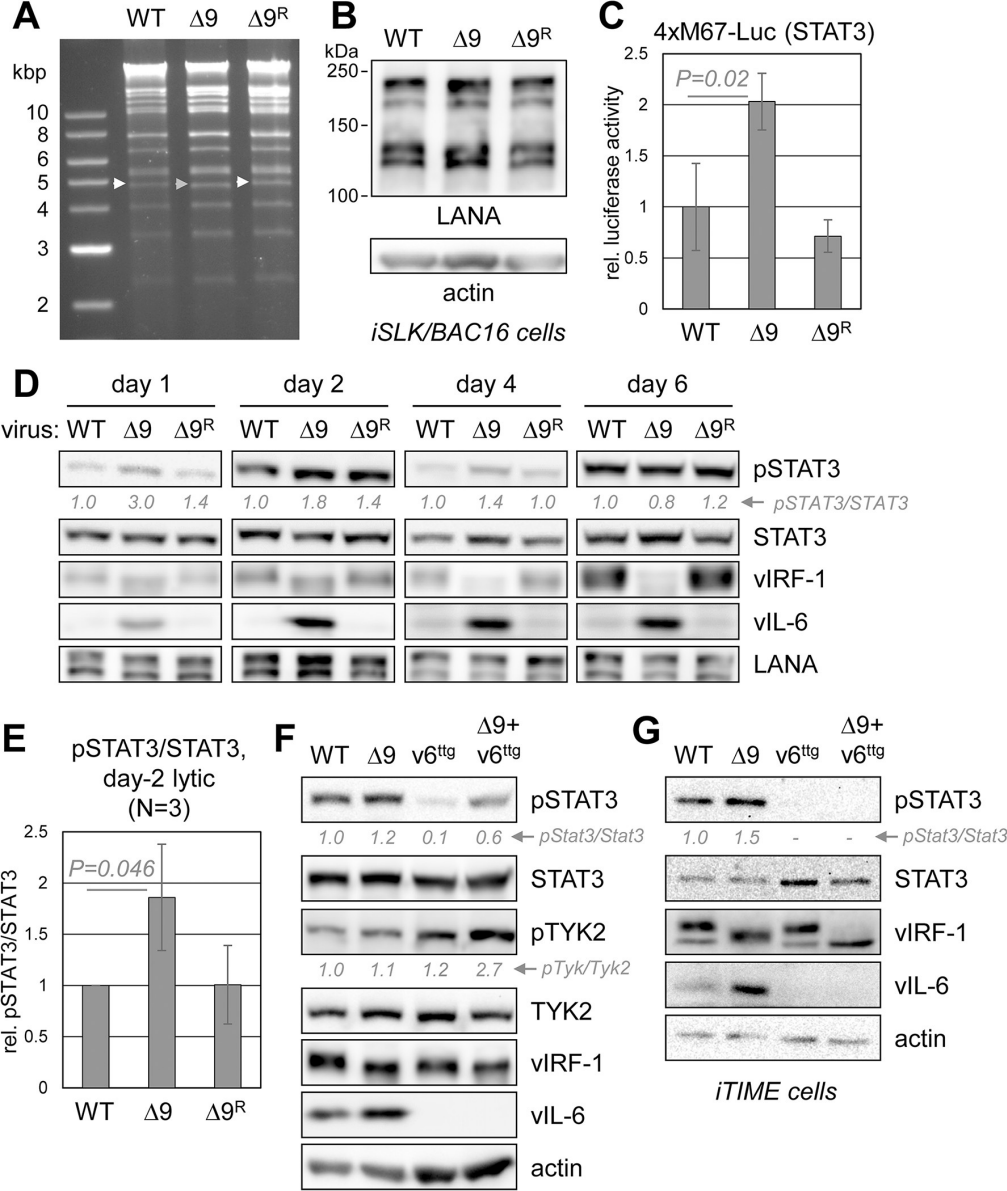
Correlation of vIRF-1-TYK2 interaction with pSTAT3 regulation in HHV-8-infected cells. (A) Restriction profiling, using SpeI endonuclease, of wild-type (WT), vIRF-1Δ9-expressing (Δ9), and Δ9-mutation-repaired (Δ9^R^) HHV-8 BAC16 genomes. The white and grey arrowheads indicate the positions of DNA fragments containing WT/repaired and Δ9 vIRF-1 ORFs, respectively. (B) LANA-immunoblot verification of equivalent latent viral loads in iSLK cultures infected with the same infectious doses of native, vIRF-1 Δ9-mutated, and repaired (control) viruses. (C) Assessment of active STAT3 in recombinant HHV-8-infected and lytically reactivated iSLK cultures using the STAT3-responsive 4xM67-Luc reporter vector, transfected into the respective cultures. Luciferase activities from triplicate cultures are expressed as means, with standard deviations indicated; the mean value for WT virus is set at 1. Significance (*P*) of the increased luciferase activity in the vIRF-1Δ9-expressing cells was determined by student’s t-test. (D) Immunoblot analysis of pSTAT3 levels in lytically reactivated iSLK cells infected with WT, Δ9, and Δ9^R^ viruses. Cells were harvested at the indicated times after Dox/NaB treatment and lysates probed for total- and phospho-STAT3, in addition to LANA and lytically-induced vIRF-1 and vIL-6. Quantified levels of pSTAT3, normalized to total-STAT3 and relative to “WT” pSTAT3 levels (set at 1), are shown below the pSTAT3 blots. (E) Combined “day-2” pSTAT3/STAT3 data from panel D and two additional experiments. Average values relative to wild-type (set at 1), standard deviations, and *P* value (student’s t-test, two-tailed) for Δ9 against wild-type virus are shown. (F) Analysis of pSTAT3 and pTYK2 levels in lysates of lytically reactivated iSLK/BAC16 cultures, incorporating vIL-6-null (v6^ttg^) and vIL-6-null/vIRF-1Δ9 (v6^ttg^+Δ9) viruses, harvested 2 days after Dox/NaB treatment. Levels of pSTAT3 and pTYK2, normalized to total-STAT3 and -TYK2 and expressed relative to levels (set at 1) in WT BAC16-infected cells, are indicated below the respective blots. (G) A similar analysis of pSTAT3 levels was undertaken using iTIME cells, harvested 2 days after lytic induction.

Having identified vIRF-1Δ9 as deficient for TYK2 interaction and STAT3-signaling suppression, we next utilized this variant for signaling analyses in infected cells. First, the Δ9 mutation was engineered into the HHV-8 BAC16 genome and a wild-type-reverted (“repaired”, Δ9^R^) genome was generated as a control, using previously described recombineering methods [35,36]. The overall integrities of the recombinant genomes (mutated and repaired) were checked by restriction analysis and comparison to the wild-type genome (Fig 7A), and the introduced changes were verified by sequencing of the appropriate genomic locus. Viruses were reconstituted from transfected and lytically-reactivated iSLK cells and titered using standard techniques (see Materials and Methods). Equal infectious doses of recombinant and wild-type viruses were used to infect iSLK cells, lysates of which were immunoblotted for latency-associated nuclear antigen (LANA) to verify equivalent viral loads (Fig 7B). These stock cultures were used experimentally. Firstly, the cultures were transfected with the STAT3-responsive reporter 4xM67-Luc to assess STAT3 activity in lytically reactivated cells. Luciferase activity was found to be significantly enhanced in the BAC16.vIRF-1Δ9 infected cells relative to cells infected with native and repaired viruses (Fig 7C). These data correlate vIRF-1-TYK2 interaction, abrogated by the Δ9 mutation, with suppression of active STAT3. Reflecting these data, immunoblotting for pSTAT3 revealed increased pSTAT3 levels in BAC16.vIRF-1Δ9 virus-infected cells relative to those infected with parental and repaired wild-type viruses at days 1, 2 and 4 after lytic reactivation (Fig 7D). Regulation of pSTAT3 as a function of the Δ9 mutation was verified by combining quantified “day-2 lytic” data from this and two further experiments (Fig 7E). Notably, disruption of vIRF-1-TYK2 interaction in BAC16.vIRF-1Δ9-infected cells led to significantly increased levels of vIL-6 (Fig 7D), which could account for or contribute to elevated pSTAT3; vIL-6 signaling via the gp130 signal transducer, intracellularly or at the cell surface, leads to activation of pSTAT3 [37–39]. Follow-up RT-qPCR analyses of mRNA from lytically infected cells identified elevated vIL-6 transcripts in BAC16.vIRF-1Δ9-infected cells relative to parental and repaired virus-infected cells (S2 Fig); this could potentially be related to IFN-I/TYK2 regulation of vIL-6 transcription via promoter-contained ISREs [40]. Our data indicate that vIRF-1-TYK2 interaction is involved in suppression of pSTAT3, and that this may involve inhibition, likely via repression, of vIL-6 expression in addition to more direct effects through inactivation of TYK2.

To dissociate vIRF-1 suppression of pSTAT3 via TYK2 from effects through vIL-6 regulation, we generated the vIRF-1 Δ9 mutation in the context of vIL-6-null BAC16 virus, in which the translation-initiation codon was changed to TTG [41]. The double-mutated BAC16 genome (BAC16.vIRF-1Δ9/vIL-6^ttg^) was checked for gross integrity by restriction analysis (S3A Fig) and reconstituted virus was titered relative to wild-type, vIRF-1Δ9 and vIL-6^ttg^ viruses in order to generate normalized infected iSLK cultures, verified by LANA immunoblotting (S3B Fig). Levels of pSTAT3 in lytically reactivated cells were elevated by the vIRF-1 Δ9 mutation either alone (as before, although modest here) or in the context of vIL-6 ablation, which caused a reduction in pSTAT3 levels (Fig 7F). Also, a clear increase in the level of pTYK2 was seen as a consequence of the Δ9 mutation in the context of vIL-6 knockout. Thus, while vIRF-1, through TYK2, may modulate pSTAT3 via regulation of vIL-6 (if not already at gp130-saturating dose), it can also do so independently of vIL-6. Increased pSTAT3 as a function of the vIRF-1 Δ9 mutation, along with increased vIL-6 expression, was detected also in lytically reactivated iTIME endothelial cells [42] (Fig 7G); here, vIL-6 ablation reduced pSTAT3 to levels that were difficult to detect.

### Phenotypic analyses of vIRF-1-TYK2 interaction and STAT3 signaling

**Fig 8:**
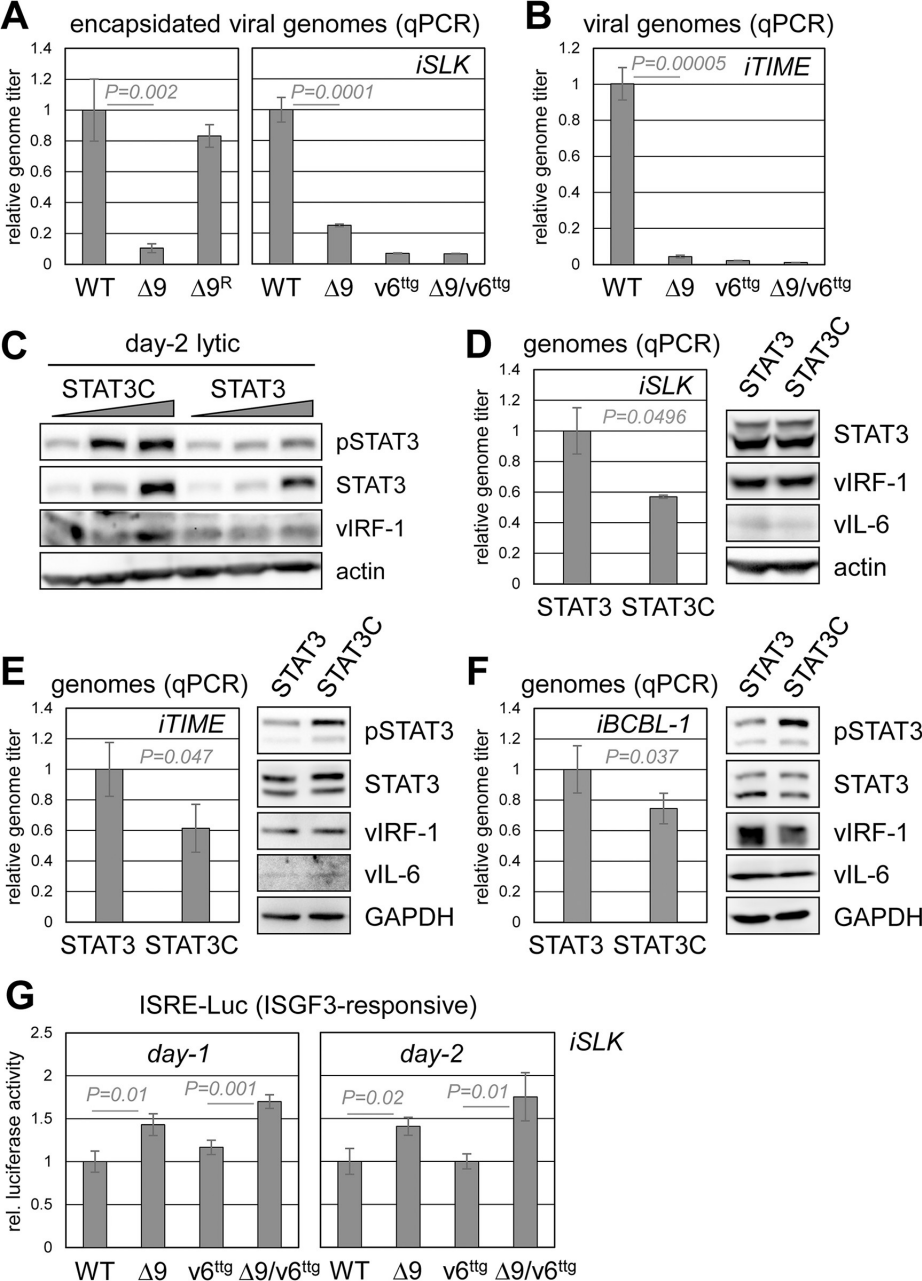
Regulation of HHV-8 replication via TYK2-interacting vIRF-1 sequences and STAT3 signaling. (A) Determinations, by qPCR, of encapsidated virus genome yields as a function of vIRF-1 Δ9 mutation (Δ9) and/or vIL-6 ablation (v6^ttg^), relative to WT virus yield, from lytically reactivated cultures (biological triplicates) over a six-day period of Dox/NaB treatment. Repaired “Δ9” virus (Δ9^R^) was used as a control. Student’s t-test-calculated *P* values are shown for “Δ9” versus “wild-type” data. (B) Analogous analyses were carried out in iTIME cells, which were induced with Dox/NaB for 4 days prior to media harvest for qPCR measurement of virus yields. (C) Assessment of pSTAT3 levels in BAC16 HHV-8-infected iSLK cells transduced with different doses of lentiviral vectors expressing CBD-tagged native STAT3 or hyper-active, dephosphorylation-resistant STAT3C [44]. The cultures were treated with Dox/NaB to induce lytic replication and harvested after 2 days for generation of cell lysates for immunoblotting. (D) Relative virus yields from 6-day-reactivated cultures were measured by qPCR. Statistical significance (*P*) of data from biological duplicates was calculated using student’s t-test. Cells were harvested for immunoblot analysis of endogenous and lentivirally-expressed STAT3 (bottom and top bands, respectively) relative to β-actin and lytically-induced vIRF-1 and vIL-6. (E) An equivalent experiment was carried out in iTIME cells, using biological triplicates. Media were harvested 4 days after lytic induction for qPCR determinations of virus production. Parallel cultures were harvested 2 days after lytic induction for immunoblot detection of total and phosphorylated native and exogenously introduced STAT3 and STAT3C along with vIRF-1 and vIL-6. (F) Analogous analyses of STAT3C effects on replication were carried out in iBCBL-1 cells; media and cells were harvested 2 days and 1 day, respectively, after lytic induction for measurement of virus production and immunoblotting for STAT3/pSTAT3 and viral proteins. (G) Testing for effects of BAC16 mutations on IFN-I signaling, reported previously to be inhibited by vIRF-1 via pSTAT1 and pSTAT2 suppression [17]. An interferon signaling response element (ISRE)-luciferase reporter was introduced into iSLK cells by lentiviral transduction, and these cells then inoculated with equal infectious doses of wild-type or vIRF-1 and/or vIL-6 ORF-mutated BAC16 viruses. Following lytic reactivation for 1 or 2 days, cells were harvested for determinations of luciferase activity. Average values from biological triplicates, relative to activity in wild-type-infected cells (set at 1), are shown along with standard deviations and student’s t-test-derived *P* values.

We next analyzed the effect of the vIRF-1 Δ9 mutation, introduced alone or in the context of vIL-6^ttg^, on HHV-8 productive replication; vIL-6-knockout virus was also used for comparison. The respective infected iSLK cultures were induced with Dox/NaB for 6 days (to recover maximum virus yields) and media harvested during this period. Relative yields of encapsidated viral genomes were established by qPCR of DNase I-resistant DNA derived from the clarified culture media, essentially as described previously [30]. The vIRF-1 Δ9 mutation and also vIL-6 ablation effected substantially reduced virus production (Fig 8A). Similar results were obtained in iTIME cells (Fig 8B). The data provide evidence that TYK2 targeting by vIRF-1 is important for lytic replication.

The signaling (Fig 7) and replication data indicate that pSTAT3 suppression by vIRF-1 may be important for HHV-8 productive replication, despite the fact that STAT3 has been demonstrated to be a positive contributor to lytic replication in the contexts of PEL and endothelial cells [27]. We hypothesized that excessive STAT3 activity, limited by vIRF-1, may inhibit replication. To test this hypothesis, we utilized a STAT3 variant, STAT3C, which contains mutations (A_661_C and N_663_C) that effect increase DNA binding and resistance to dephosphorylation and thereby greater and prolonged activity [43,44]. STAT3C or wild-type STAT3 were introduced into iSLK cells via lentiviral vector infection, using different doses of the respective lentiviruses, and STAT3 expression and phosphorylation were monitored 2 days after lytic induction. As expected, there was increased phosphorylation of STAT3C relative to native STAT3 (adjusted for STAT3/C expression) (Fig 8C). Cultures of iSLK cells infected with doses of the lentiviruses appropriate to yield equal STAT3 and STAT3C expression were then used to determine virus yields from the respective HHV-8^+^ cultures. Relative levels of media-contained encapsidated viral genomes, quantified by qPCR, revealed reduced titers from the STAT3C-expressing cells (Fig 8D). Immunoblotting of cell lysates (Fig 8D, right) verified equal expression of STAT3 and STAT3C; there were also similar levels of vIL-6 expression, indicating that STAT3 signaling, enhanced by STAT3C, does not contribute to vIL-6 induction associated with the vIRF-1 Δ9 mutation (Fig 7D). Suppression of productive replication by STAT3C was observed also in iTIME cells (Fig 8E) and in iBCBL-1 PEL cells (Fig 8F). The suppression of virus production by STAT3C demonstrates negative effects on lytic replication of abnormally high STAT3 activity.

Whilst vIRF-1 regulation of pSTAT3, through TYK2 inactivation, could help establish intracellular conditions optimal for HHV-8 replication, vIRF-1 activity via TYK2 likely includes the suppression of pSTAT2 and IFN-I signaling [17]. Indeed, using an ISRE reporter in infected iSLK cells, we were able to demonstrate significantly increased IFN-I signaling as a function of introduced vIRF-1 Δ9 mutation, alone or together with vIL-6 ablation, which itself had little effect (Fig 8G). Thus, loss of vIRF-1 interaction with TYK2 could affect HHV-8 replication also via increased antiviral IFN-I signaling.

### Phenotypic analyses of vIRF-1-STAT3 interaction

**Fig 9:**
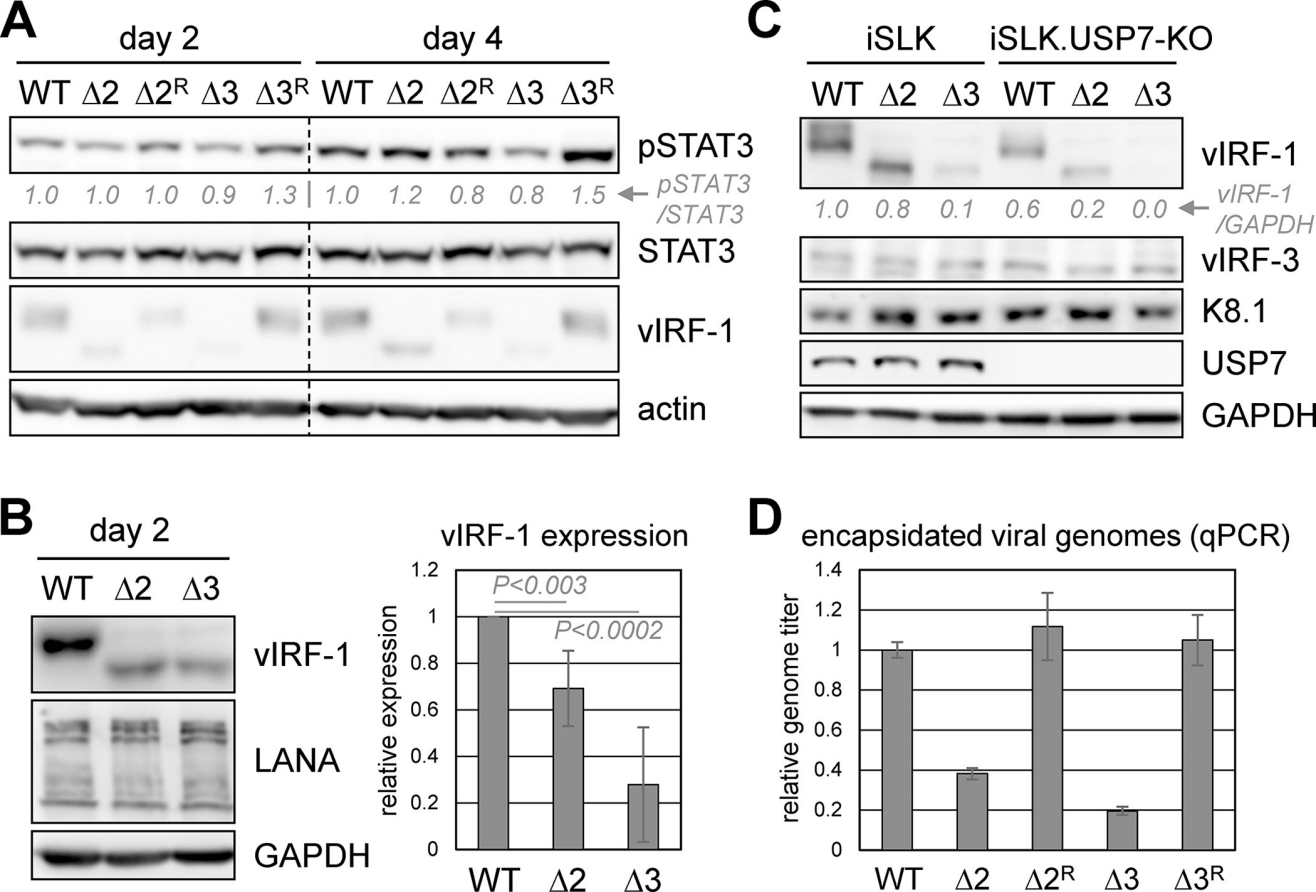
Genetics-based analyses of effects of vIRF-1-STAT3 interaction. (A) BAC16-based wild-type (WT) and engineered HHV-8 viruses expressing vIRF-1Δ2 (Δ2) or vIRF-1Δ3 (Δ3) and wild-type-reverted derivatives (Δ2^R^, Δ3^R^) were used to establish latent infections in iSLK cells. The cultures were treated with Dox/NaB to induce lytic replication and harvested after 2 and 4 days for immunoblot detection of pSTAT3 levels, along with total-STAT3 and vIRF-1. Levels of pSTAT3, relative to “WT” levels and normalized to total-STAT3, are shown below the pSTAT3 blot. (B) Comparisons of vIRF-1Δ2 and vIRF-1Δ3 expression relative to wild-type vIRF-1 in BAC16-virus-infected iSLK cells, harvested 2 days after lytic induction. The chart (right) shows quantified data from 5 independent experiments, with average values expressed relative to wild-type vIRF-1 levels. (C) Similar analysis of wild-type, Δ2 and Δ3 vIRF-1 expression in USP7-knockout iSLK cells, in comparison to native iSLK cells, and co-detection of other lytic proteins. Relative levels of vIRF-1 expression, normalized to GAPDH, are shown below the vIRF-1 blot. (D) Assessment of replication competence of the recombinant viruses, relative to wild-type HHV-8, by qPCR of encapsidated genomes released into iSLK culture media over six days of Dox/NaB treatment. Data were derived from triplicate cultures; average values and standard deviations between replicates are shown.

To investigate potential effects of vIRF-1-STAT3 interaction on signaling and productive replication in iSLK cells, we generated BAC16-based HHV-8 mutants that contained either the Δ2 or Δ3 deletions in vIRF-1, each of which diminishes vIRF-1 interaction with STAT3 (Fig 3A), and, as controls, repaired versions thereof. The bacmid genomes were checked by restriction digestion for gross integrity, relative to the wild-type genome (S3C Fig), and normalized infectious doses were used for parallel infections of iSLK cultures, lysates of which were checked for equivalent levels of LANA expression following latency establishment and hygromycin-selection (S3D Fig). These cultures were treated with Dox/NaB for lytic reactivation and cultures harvested at 2 and 4 days for immunoblot analyses. Generally consistent with transfection-derived data (Fig 3), pSTAT3 levels were not changed substantially as a result of the vIRF-1 Δ2 or Δ3 mutation (Fig 9A), indicating that physical targeting of STAT3 does not influence levels of active STAT3 in infected iSLK cells. Interestingly, levels of vIRF-1 appeared to be somewhat lower in cells infected with mutant viruses relative to native and wild-type-reverted (repaired) viruses. Further analyses of vIRF-1 levels in lytically reactivated iSLK cultures verified that vIRF-1Δ2 and vIRF-1Δ3 were expressed at reduced levels relative to native vIRF-1 (Fig 9B).

Of note, each of the introduced mutations ablated a USP7-binding motif spanning the junction between the Δ2 and Δ3 regions [45]. This could potentially affect vIRF-1 expression, conceivably regulated via ubiquitination-directed proteasomal degradation and stabilized by USP7-mediated deubiquitination. Therefore, we carried out further vIRF-1 expression analysis in USP7-null iSLK cells (engineered using CRISPR). Immunoblotting of day-2 lytic-cell lysates revealed diminished expression of vIRF-1Δ2 and vIRF-1Δ3 relative to wild-type vIRF-1 in USP7-null cells as in native iSLK cells (Fig 9C), thereby correlating vIRF-1 expression with STAT3 interaction, independently of USP7 targeting. However, expression of vIRF-1 (but not other lytic proteins examined) was reduced by USP7 ablation, indicating that vIRF-1 may be a substrate of the deubiquitinase and regulated via ubiquitination. With respect to replication, while the mutations led to diminished yields of qPCR-measured encapsidated viral genomes in iSLK cultures (Fig 9D), attempts to detect USP7-independent replication effects of the Δ2 and Δ3 mutation in USP7-knockout cells was not possible due to severely reduced virus production in these cells. The USP7-binding motif disrupted by the Δ2 and Δ3 mutations is known to be required for optimal HHV-8 lytic replication in iSLK cells [30].

**Fig 10:**
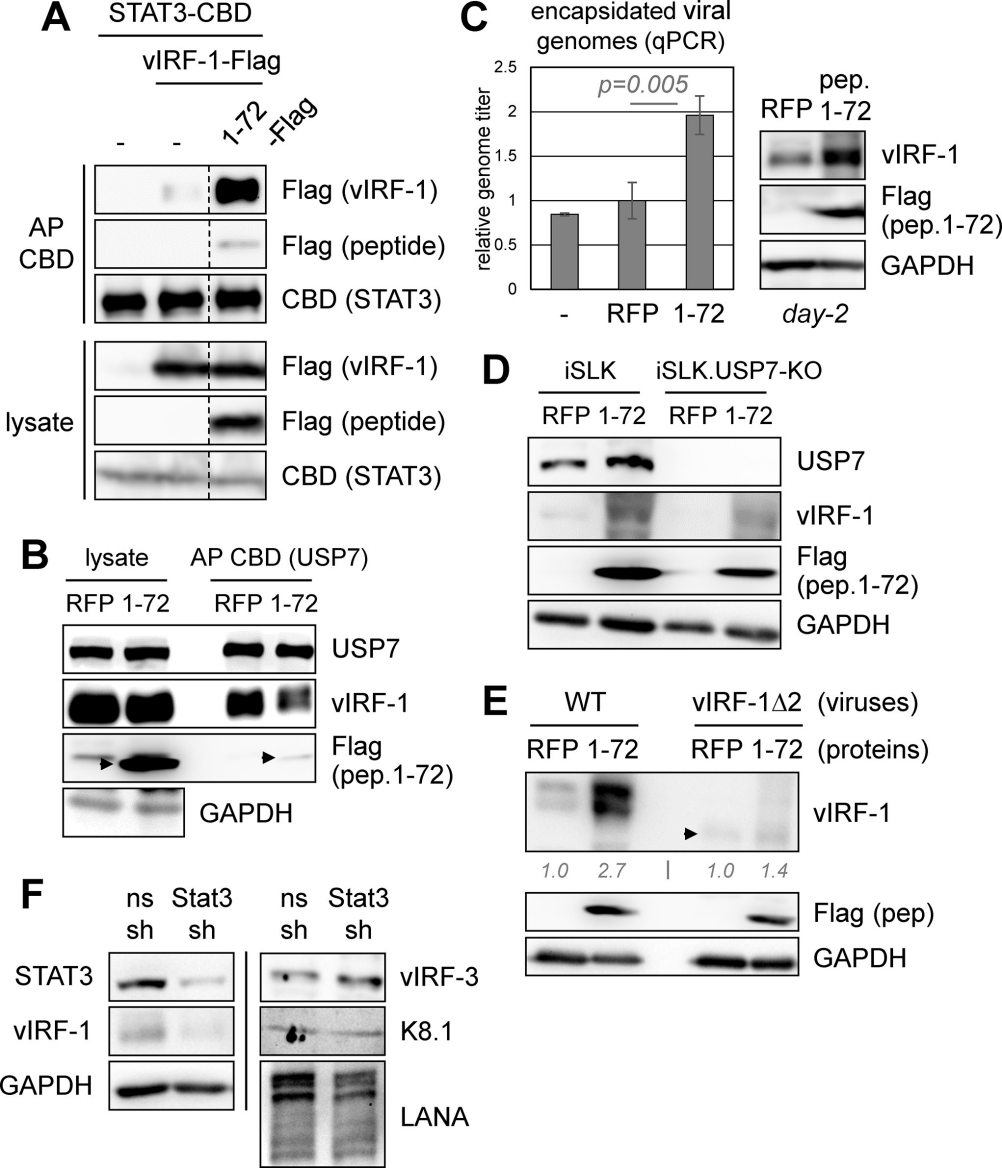
Effects on virus replication and vIRF-1 expression of peptide-mediated vIRF-1:STAT3 manipulation. (A) Testing of a peptide comprising Flag-tagged vIRF-1 residues 1–72 on vIRF-1-STAT3 interaction in STAT3-CBD and vIRF-1-Flag vector-transfected 293T cells, using STAT3-CBD affinity precipitation. Immunoblotting of cell lysates confirmed equivalent expression of vIRF-1 and expression of peptide 1–72 in the respective samples. The dotted line indicates deletion of a lane. (B) Analogous assessment of peptide 1–72 influence on vIRF-1 interaction with USP7. (C) Effect of lentiviral vector-expressed peptide 1–72, relative to no-peptide (-) or RFP controls, on HHV-8 productive replication, as measured by qPCR of cell-released encapsidated (DNase I-resistant) viral genomes following 4 days of Dox/NaB treatment. Data are from biological triplicates; average values, relative to RFP value (set at 1), are shown along with standard deviations and statistical significance (student’s t-test). The identified positive effect of peptide 1–72 on virus production was accompanied by immunoblot-detected increased vIRF-1 expression (in day-2-harvested parallel cultures). (D) Testing of peptide 1–72, relative to control RFP, for enhancement of vIRF-1 expression in USP7-knockout iSLK cells (iSLK.USP7-KO), harvested 2 days after lytic induction. Wild-type iSLK cells were used as a positive control. (E) Assessment of susceptibility of STAT3-refractory vIRF-1Δ2 to regulation by lentiviral vector-expressed peptide 1–72, relative to RFP (negative control), in Dox/NaB-treated iSLK cells infected with either wild-type BAC16 or BAC16.vIRF-1Δ2 virus. Cells were harvested 2 days after lytic induction for immunoblot analysis of cell extracts. Levels of vIRF-1 and vIRF-1Δ2 (arrowhead) in peptide-expressing cells relative to RFP^+^ controls (normalized to GAPDH levels) are shown below the vIRF-1 blot. (F) Correlation of STAT3 depletion with vIRF-1 suppression, absent effects on other viral proteins, in lytically reactivated HHV-8^+^ iSLK cells treated with Dox/NaB for 48 h before cell harvest and immunoblotting of cell lysates. Non-silencing (ns) shRNA (sh) was used as a control.

To assess the effects of vIRF-1-STAT3 interaction by an alternative approach, we generated a lentivirus vector for expression of a putative STAT3-interaction-disruptive peptide derivative of vIRF-1 N-terminal residues 1 to 72 (spanning the Δ1-Δ3 region) implicated in STAT3 interaction. Transfection-based affinity precipitations of STAT3-CBD determined that the peptide (pep.1-72) greatly enhanced binding (Fig 10A), rather than inhibiting the interaction via expected competitive interaction. The peptide itself was precipitated by STAT3-CBD, demonstrating that interaction between vIRF-1 and STAT3 is, at least in part, mediated via these sequences. That this peptide can enhance vIRF-1-STAT3 binding indicates that it may compete with a cellular inhibitor of the interaction; this will require further investigation. Of note, pep.1-72, when used in a 4xM67-Luc reporter assay, had little effect on vIL-6-induced STAT3 activity in vIRF-1-coexpressing cells (S4 Fig), indicating that the vIRF-1 interaction with STAT3 does not affect pSTAT3 binding to the reporter *cis*-elements or pSTAT3 transcriptional activity. In a coprecipitation assay of vIRF-1-USP7 interaction, pep.1-72 competed for binding and associated with USP7 (Fig 10B), as expected. To assess potential activity of pep.1-72 in HHV-8 replication, HHV-8^+^ iSLK cells were infected with peptide-expressing lentivirus or RFP-expressing control vector or left untreated, lytic replication induced, and cumulative virus yields over 6 days assessed by qPCR, as before. Peptide-expressing cells yielded increased virus production relative to untreated and RFP-expressing cells (Fig 10C), over and above any anti-replicative effect of the peptide effected via disruption of vIRF-1-USP7 interaction [30]. Notably, immunoblot analysis of cell extracts from parallel (day-2) lytic cultures detected substantially increased vIRF-1 expression in the peptide-expressing cells (Fig 10C, right). Further investigation determined that this was not caused by a general increase in viral gene expression (S5 Fig). As vIRF-1 has been reported to act as a positive factor in productive replication in this cell type [10,30], in addition to PEL and endothelial cells [11,30,46], its increased expression may account for or contribute to the observed enhanced virus production in peptide-expressing cells. Introduction of pep.1-72 into USP7-knockout iSLK cells also led to increased vIRF-1 expression (Fig 10D), demonstrating USP7-independent regulation by the peptide. Consistent with positive regulation of vIRF-1 by pep.1-72 via promotion of vIRF-1-STAT3 interaction, STAT3-refractory vIRF-1Δ2 was resistant to regulation by the peptide (Fig 10E). Although the data do not distinguish between direct effects on HHV-8 productive replication of pep.1-72-enhanced vIRF-1-STAT3 interaction and indirect effects via vIRF-1 expression, the presented peptide- and genetics-based analyses of vIRF-1 targeting of STAT3 indicate an important biological role of the interaction via positive regulation of vIRF-1 expression. Further supporting a role of STAT3 in vIRF-1 regulation, STAT3 depletion led to substantially reduced levels of vIRF-1, but not other tested viral proteins (Fig 10F).

Further experiments utilizing pep.1-72 and STAT3 depletion were carried out in iTIME and iBCBL-1 cells. Expression of pep.1-72 in iTIME cells led to increased replication and vIRF-1 expression (S6A Fig), as in iSLK cells. In iBCBL-1 cells, the peptide increased virus replication but not vIRF-1 expression (S6B Fig), indicating pro-replicative effects of vIRF-1-STAT3 interaction independently of vIRF-1 regulation. This may occur also in iSLK and iTIME cells, in addition to effects mediated via vIRF-1 regulation. STAT3 depletion in iBCBL-1 and iTIME cells led to diminished vIRF-1 expression (S6C and S6D Fig), demonstrating a role of STAT3 in vIRF-1 regulation in these cells as in iSLK cells.

## Discussion

STAT3 has been reported to be a positive factor in productive replication for several viruses. For example, hepatitis B and C viruses (HBV, HCV) promote STAT3 activation, indirectly and via interactions of HBV X protein with STAT3-activating JAK1 kinase and direct interaction of the HCV core protein with STAT3, and this is important for productive replication [47–52]. Among herpesviruses, human cytomegalovirus (HCMV) can activate STAT3 through several mechanisms that induce STAT3-activating interleukin-6 (IL-6) and also via viral IL-10-induced STAT3 phosphorylation, but although STAT3 is important for virus replication, HCMV can attenuate activating phosphorylation of STAT3 via viral IE1 protein-mediated sequestration of unphosphorylated STAT3 in the nucleus [53–55]. For varicella-zoster herpesvirus, STAT3 activity plays a positive role in productive replication, in part, at least, by the induction of pro-survival factor survivin [56].

Reported roles of STAT3 in HHV-8 biology include promotion of PEL-cell viability, suppression of latent-lytic switching in PEL cells, and promotion of reactivated productive replication in PEL and endothelial cells [25–27,29]. Understanding the viral mechanisms that regulate STAT3 activation and signaling is therefore important. One major mechanism of STAT3 activation by HHV-8 is via vIL-6/g130 signaling. This occurs in latently infected PEL cells and contributes to cell growth and viability and promotes virus productive replication in PEL and endothelial cells and likely also in iSLK cells, where vIL-6 and its interaction with gp130 promote HHV-8 replication [26–28]. Like vIL-6, vIRFs 1, 2 and 3 are expressed in latently infected PEL cells, in addition to lytically infected cells, and contribute to PEL cell viability and modulate productive replication, both positively (vIRF-1) and negatively (vIRFs 2 and 3) [30,46,57,58]. Here, we have shown that vIRF-1 can regulate STAT3 activation, suppressing levels of active pSTAT3 and downstream STAT3 signaling in both transfected and HHV-8-infected cells. Opposing the effects of vIL-6 with regard to STAT3 regulation, it is possible that vIRF-1 serves to restrict pSTAT3 levels within limits that are biologically advantageous. Indeed, our studies of lytic replication in iSLK, iTIME and iBCBL-1 cells expressing hyper-active STAT3 variant STAT3C demonstrated that excessive STAT3 activation led to reduced virus yields (Fig 8D–8F). Experiments utilizing a virus expressing TYK2-refractory vIRF-1Δ9 protein in place of wild-type vIRF-1 revealed mutation-effected increases in pSTAT3 levels during lytic replication in iSLK and iTIME cells (Fig 7) and a marked suppression of virus production associated with abrogation of TYK2 interaction (Fig 8A and 8B). While the Δ9 mutation in vIRF-1 blocked vIRF-1 interaction with and inhibition of TYK2, expected to effect increased STAT3 phosphorylation, it also led to increased expression of STAT3-activating vIL-6. Increased pSTAT3 levels in iSLK cells infected with vIL-6-null virus containing the vIRF-1 Δ9 mutation showed clearly that this mutation could induce STAT3 activation independently of vIL-6, demonstrating the specific significance of vIRF-1-TYK2 interaction for modulation of STAT3 signaling (Fig 7F). With respect to regulation of vIL-6 by vIRF-1, it is notable that a previous study reported that vIRF-1 antisense RNA effected markedly reduced expression of vIL-6, in addition to viral early lytic genes PAN and K8, in lytically reactivated PEL (BCBL-1) cells, while another study reported interaction of vIRF-1 with *cis*-elements of the K3-ORF promoter, upstream of the vDHFR (ORF2) and vIL-6 (ORF-K2) genes, and repression of K3-promoter-driven transcription in a reporter assay [59,60]. Although the latter report suggested potential transcriptional repression of vIL-6 by vIRF-1, no evidence of such a mechanism was provided. Our data indicate that in the contexts of iSLK and iTIME cells, vIL-6 is regulated negatively by vIRF-1 via TYK2 interaction (potentially through suppression of IFN-I signaling [40]), but it is conceivable that other interactions and/or indirect effects of vIRF-1 mediate positive regulation of the viral cytokine.

Our presented data identify direct vIRF-1 interaction with STAT3, in addition to TYK2, and involvement of N-terminal sequences of vIRF-1. Thus, deletion variants Δ1, Δ2 and Δ3 of vIRF-1 were diminished for interaction with STAT3 (Fig 3A). This allowed for genetics-based analyses of the interaction with respect to pSTAT3 regulation. Consistent with results from transfection experiments, abrogation of vIRF-1 interaction with STAT3, by introduction of Δ2 or Δ3 vIRF-1 mutations into HHV-8 BAC16, had little or no effect on pSTAT3 levels in lytically infected iSLK cells. However, it had a marked negative effect on virus yields and corresponded with decreased expression of vIRF-1. It is notable that a previous report utilizing a vIRF-1 variant deleted of the Δ1 to Δ3 region (residues 1–72) of vIRF-1 was defective for “rescue” of HHV-8 productive yields in PEL (TRExBCBL1-RTA) cells depleted of virus-produced vIRF-1, and this was correlated with deficient suppression of apoptotic signaling and pro-viability activity of vIRF-1 [46]. This region of vIRF-1 contains sequences (residues 32–77) involved in, and able to confer, mitochondrial localization and also residues (N8 and F10) required for interaction of vIRF-1 with NIX, involved in vIRF-1-promoted mitophagy [46,61]. While our vIRF-1 Δ2 (residues 23–47) and Δ3 (residues 48–72) mutations would not be expected to affect NIX interaction, each may alter mitochondrial localization and associated pro-viral functions, albeit that each of these regions contains putative, possibly redundant, mitochondrial localization signals. Therefore, replication-suppressive effects of the mutations via inhibition of mitochondrial-associated activities, rather than or in addition to effects via STAT3 targeting, cannot be excluded. Similarly, the Δ2 and Δ3 mutations of vIRF-1 alter a USP7-binding motif shown previously to play a positive role in HHV-8 replication [30]. However, the inverse regulation of vIRF-1 levels by the STAT3-binding-disrupting vIRF-1 Δ2/Δ3 mutations and vIRF-1-STAT3 interaction-promoting pep.1-72 (both of which disrupt USP7 binding) implicates vIRF-1-STAT3 interaction, specifically, with enhanced expression of vIRF-1 in iSLK cells. Furthermore, HHV-8 replication was enhanced by pep.1-72 despite the known suppressive effect on replication of disruption of USP7 targeting by vIRF-1 [30], again indicating an important biological role specifically of STAT3 targeting by the viral protein. The 72-residue peptide also increased virus replication and vIRF-1 expression in iTIME cells. It is notable, however, that while pep.1-72 greatly enhanced virus production in iBCBL-1 cells, as in iSLK and iTIME cells, it did so absent increased vIRF-1 expression, dissociating the two activities and indicating an independent mechanism of vIRF-1-STAT3 interaction-promoted replication in this cell type. Further studies will be required to determine the underlying molecular basis and full biological significance of STAT3 binding by vIRF-1, including the possibility that it might modulate the specificity of promoter-targeting by the transcription factor.

Data presented here are the first to identify direct interaction of vIRF-1 with TYK2, and verify, using various approaches, our previously reported inhibition of TYK2 activity by vIRF-1. In our previous study [17], TYK2 inhibition by vIRF-1 was linked to suppression of levels of active (phosphorylated) STAT2, involved, along with STAT1, in type-I interferon signaling. Through the use of an IFN-I-responsive ISRE-luciferase reporter and viral mutants in lytically-reactivated iSLK cells, here we were able to identify definitively the importance of vIRF-1-TYK2 interaction for the suppression of IFN-I signaling (Fig 8G). Interestingly, although STAT3 can negatively regulate IFN-I signaling [18], vIL-6 ablation, leading to reduced pSTAT3, had no effect on ISRE-reporter activity or its increase by the vIRF-1 Δ9 mutation, indicating that the detected effect of the mutation was mediated primarily via increased pSTAT2 and pSTAT1 [17]. Thus, reduced virus titers effected by the vIRF-1 Δ9 mutation may result from enhanced antiviral IFN-I signaling in addition to increased (non-optimal) pSTAT3 levels, and indeed other mechanisms may be involved.

Other reported viral targeting of TYK2 include that by latency membrane protein-1 (LMP-1) and BGLF2-encoded tegument protein of Epstein-Barr virus (EBV) [62,63], human papillomavirus (HPV) E6 protein [64], and various paramyxovirus V proteins [65,66], multiple members of the latter also interacting with JAK1. The V proteins mediate suppression of interferon signaling involving IFN receptor-associated TYK2 and JAK1. Interestingly, some Morbillivirus-genus paramyxovirus V proteins interact with STAT1 and STAT2 in addition to TYK2, but inhibition only of IFN-II signaling, not that by IFN-I, was associated with interaction with STAT1 (activated predominantly by IFN-II) [65]. Somewhat contrastingly, we found that TYK2 interaction, rather than binding to STAT3, is the key mechanism of STAT3 suppression by vIRF-1. For HPV E6, interaction with TYK2 was found to be direct and via residues of the kinase required for its interaction with IFN-I receptor 1 (IFNAR1), indicating that the mechanism of E6 suppression of IFN-I-induced TYK2 activation (phosphorylation) and downstream activation of STAT1 and STAT2 is mediated via disruption of IFNAR1 association with TYK2 [64]. We have reported previously that vIRF-1 inhibits IFN-I signaling via STAT1-STAT2 disruption, TYK2 inhibition, and disruption of TYK2 and STAT2 interactions with IFNAR1 [17]. In the previous and present studies, our data show that vIRF-1 is able to inhibit TYK2 autophosphorylation effected by TYK2 overexpression (independently of IFN signaling), indicating direct inhibition of the kinase by vIRF-1. Although the TYK2 residues targeted by vIRF-1 are not known, we show here that the interaction is necessary for TYK2 inhibition, and, considering the presently demonstrated bimolecular interaction between vIRF-1 and TYK2, likely sufficient. It is notable that EBV LMP-1 inhibits RSV-activated TYK2 phosphorylation via a mechanism requiring a specific TYK2-binding motif in the C-tail of the receptor [62], but it is unclear if LMP-1 can directly inhibit TYK2 kinase activity.

**Fig 11:**
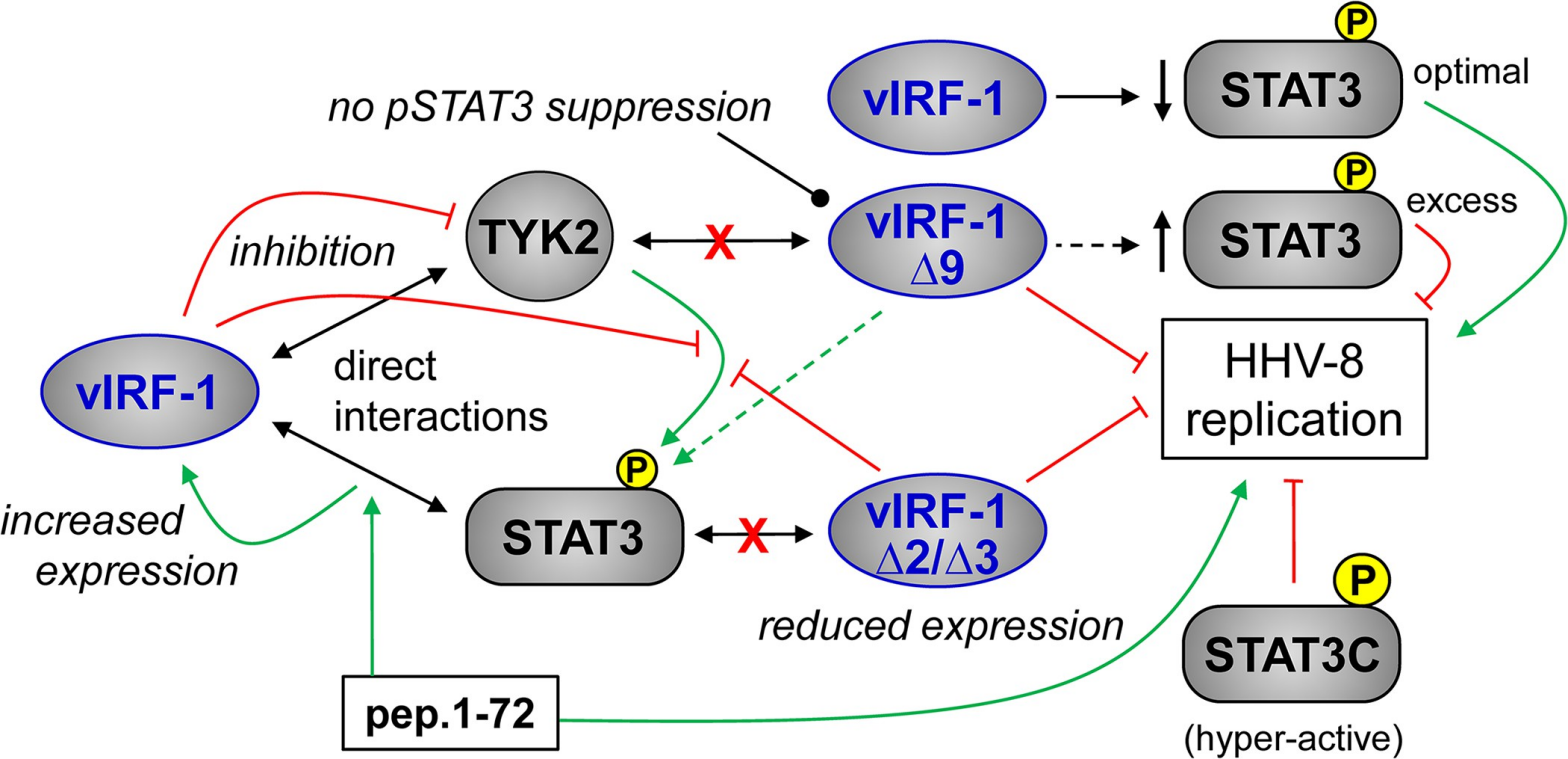
Summary of main findings. Data presented here have revealed direct interaction of vIRF-1 with STAT3 (phosphorylated and unphosphorylated) and its kinase TYK2. Abrogation of TYK2 interaction by the Δ9 mutation in vIRF-1 led to loss of suppression by vIRF-1 of active, phosphorylated STAT3 (pSTAT3) whereas STAT3 targeting, disrupted by vIRF-1 Δ2 and Δ3 mutations, had no effect. However, the latter interaction correlated with normal expression of vIRF-1, which was reduced in infected cells by the Δ2 and Δ3 mutations (effecting greatly diminished virus replication) and enhanced by peptide 1–72 (pep.1-72) that promotes vIRF-1-STAT3 interaction. Also, STAT3 depletion in infected cells led to reduced expression of vIRF-1 during lytic replication (not indicated). While the Δ9 mutation diminished virus productive replication in recombinant virus-infected cells, pep.1-72 promoted virus production. Levels of pSTAT3 in recombinant vIRF-1Δ9-virus-infected cells supporting lytic reactivation were higher than in cells infected with wild-type virus or vIRF-1Δ2 or vIRF-1Δ3 viruses. Introduction of the hyper-active, dephosphorylation-resistant STAT3C variant into infected cells inhibited productive replication, indicating that excessive pSTAT3 is detrimental to virus replication. Overall, the presented data show that vIRF-1 can suppress levels of active STAT3 via interaction with and inhibition of TYK2, likely helping to achieve levels of pSTAT3 compatible with efficient virus replication, while vIRF-1 interaction with STAT3 can effect positive regulation of vIRF-1, a viral protein known to support productive replication, and promote replication via this and/or other mechanisms.

In summary, we have identified direct interactions of vIRF-1 with both TYK2 and STAT3, the importance of the former interaction for inhibition of TYK2 activity, suppression of active-STAT3, and promotion of HHV-8 productive replication, and involvement of vIRF-1-STAT3 interaction in the positive regulation of vIRF-1 expression and virus replication (Fig 11). These findings extend our understanding of vIRF-1 interactions and activities in HHV-8 biology.

## Materials and method

### Cell culture and transfections

HEK-293T cells and Dox-inducible and RTA-expressing iSLK cells [33] were cultured in Dulbecco-modified Eagle’s medium (DMEM) supplemented with 10% fetal bovine serum (FBS), 10 μg/ml gentamycin, and 2.5 μg/ml plasmocin (Invitrogen). TRExBCBL1-RTA cells [31] were grown in RPMI-1640 medium with the same FBS and drug supplements. Endothelial TIME-TRE/RTA (iTIME) cells expressing RTA in a Dox-dependent manner [42] were growth in EGM-2 MV medium (Lonza, #CC-3202), containing 5% FBS and cytokine supplements. USP7-knockout iSLK cells were derived by transduction with a Cas9- and gRNA-expressing lentivirus vector (see below) and selected in blasticidin (10 ng/ml) for ~10 days, prior to experimental use. For routine transfections, 293T cells were seeded one day before transfection and at 60–70% confluence were transfected with mixtures of plasmid DNA and cationic polymer liner polyethylenimine (Polysciences, #593215), as reported previously [67]. For transfection of luciferase-expressing reporter and other plasmids into iSLK cells, media of cultures at 70% confluence were replaced with fresh Opti-MEM (Gibco, #31985062), then were transfected with mixtures of the plasmids and Lipofectamine 2000 (Thermo Fisher, #11668030). After incubation for at least 6 h, the inoculum was replaced with fresh medium (DMEM containing 10% FBS); luciferase assays were performed 24 hours later. For generation of vIL-6 conditioned medium, pSG5-vIL-6 [68] was transfected into 293T cells; empty pSG5 vector (control) was transfected into parallel cultures. Two days after transfection, culture media were collected, briefly centrifuged, and then passaged through 0.45-μm filters to remove any particulates. The cleared media were aliquoted and frozen at -80°C until use.

### Plasmids

Lentiviral vector-based expression plasmids for StrepII+Flag (SF)-tagged vIRF-1 and vIRF-2 and chitin-binding domain (CBD) or S protein-fused TYK2 have been reported previously [17]. Vectors expressing ~25mer deletion variants of vIRF-1, generated in this laboratory by Dr. G. Sandford, were reported previously by Dr. Y.B. Choi [46]. Lentiviral plasmid vectors expressing CBD-tagged STAT3 and STAT3C were generated by replacing RFP coding sequences in pCEBZ-RFP-CBD [69] with NotI- and XhoI-flanked STAT3- and STAT3C-coding sequences. STAT3 sequences were PCR-amplified from a cDNA library (prepared in-lab from TRExBCBL1-RTA cells) using appropriate custom primers; coding sequences specifying STAT3C (containing A_661_C and N_663_C amino acid substitutions) were generated using corresponding mutagenic PCR primes applied to native-STAT3 coding sequences. An expression vector for CBD-fused STAT1 was generated by cloning of STAT1-encoding sequences, derived by PCR amplification of cDNA, between the NotI and XhoI restriction sites of pCEBZ-RFP-CBD (replacing RFP). A lentiviral vector expressing Flag-tagged vIRF-1 residues 1–72 was made by cloning corresponding, PCR-amplified ORF sequences, flanked by NotI and BamHI restriction sites, into pCEBZ-vIRF1-SF (replacing vIRF-1). For generation of USP7-knockout iSLK cells, the lentiviral vector lentiCRISPRv2-blast (Addgene #98293; deposited by Dr. B Stringer) was used to express a USP7 gene-complementary gRNA, with the target sequence GAGTGATGGACACAACACCG. A double-stranded DNA oligonucleotide corresponding to the gRNA sequence was inserted between the two BsmBI cloning sites in the lentiviral vector. Lentiviral vectors expressing shRNAs were used for depletion of specific transcripts and encoded proteins. A vector expressing non-silencing (NS) control shRNA was described previously [39]. Equivalent vectors expressing shRNAs targeting JAK1 (shRNA-1 and -2), TYK2 (shRNA-2 and -3), and STAT3 (shRNA-2) were cloned between the BamHI and MluI sites of lentiviral vector pYNC352, containing the H1 polIII promoter for shRNA expression and a CMV promoter-driven GFP expression cassette. The target sequences for the shRNAs were as follows: JAK1-shRNA-1: CTGAGCTACTTGGAGGATAAA; JAK1-shRNA-2: GCACGAGAACACACATCTATT; TYK2-shRNA-2: GAGTGCCTGAAGGAGTATAAG; TYK2-shRNA-3: GCAGATGGTCATGGTCAAATA; STAT3-shRNA2: GGCGTCCAGTTCACTACTAAA. GST-tagged vIRF-1 was expressed in bacteria using a vector that was reported previously [11]. A prokaryotic expression vector expressing His_6_-tagged STAT3 was generated by insertion of PCR-generated, Nde- and SalI-flanked coding sequences into the corresponding cloning sites of pET22b(+) (Novagen, #69744). Luciferase reporter plasmid 4xM67 pTATA TK-luc was provided by Dr. J. Darnell (Addgene, #8688), pRL-TK, expressing Renilla luciferase constitutively, was obtained from Promega (#E2241), and pSBE4-Luc was provided by Dr. B Vogelstein (Addgene, #16495).

### Lentivirus production

Lentivirus stocks were generated by cotransfecting ~80%-confluent 293T cultures in 75-cm^2^ flasks with 12 μg of lentiviral vector DNA and 9 μg and 3 μg, respectively, of psPAX2 and pMD2.G packaging plasmids (Addgene #12260 and #12259, deposited by Dr. Didier Trono). After incubation for at least 6 h, the transfection medium was replaced with fresh medium and the cells were cultured for a further 60 h. Lentivirus-containing medium was harvested and virus pelleted by ultracentrifugation at 25,000 rpm in a SW32Ti rotor for 2 h at 4°C. Pelleted virus was resuspended in 4 ml of DMEM containing 10% FBS and stored in aliquots at -80°C.

### Immuno- and affinity-precipitations

Transfected 293T cells expressing Flag-, CBD-, or S-tagged proteins were harvested 48 h post-transfection and lysed in lysis buffer (50 mM Tris-HCl [pH7.5], 150 mM NaCl, 5 mM EDTA, 0.2% NP40) containing protease inhibitor cocktail (Sigma, P8340) for at least 30 min. at 4°C, the lysate sonicated (using a micro-tip) for nuclear disruption, and the extract clarified by centrifugation at 15,000 x g for 15 min at 4°C. The supernatants were used in subsequent coprecipitation assays, using Flag-antibody beads (Sigma, M8823), chitin resin (New England Biolabs, S6651) or S-protein beads (Novagen, 69704), according to the manufacturer’s instructions. Incubations were carried out at 4°C overnight, with gentle shaking. Bound precipitates were washed three to five times with NP40-containing wash buffer (50 mM Tris-HCl [pH 7.5], 150 mM NaCl, 0.04% NP40). Coprecipitates were released by adding SDS-PAGE loading buffer and boiling at 95°C for 10 min. prior to gel fractionation and immunoblotting.

For *in vitro* coprecipitation assays, BL21(DE3) cells transformed with pET22b(+)- or pGEX-4T-1-based vectors (see above) were used to generate His_6_- or GST-linked proteins; expression was induced by addition of 1 mM isopropyl-β-D-thiogalactopyranoside (IPTG) for 24 h at 17°C. Pelleted cells were resuspended in lysis buffer (300 mM NaCl, 10 mM imidazole, 50 mM NaH_2_PO_4_ [pH 8.0]), for His_6_-tagged protein, or PBS (137 mM NaCl, 2.7 mM KCl, 10 mM Na_2_HPO_4_ [pH 7.4]), for GST fusions, containing 1 mg/ml lysozyme. Lysate samples, kept in an ice-water mixture, were sonicated with a micro-tip until the lysate was transparent and were cleared by centrifugation at 7,000 x g for 30 min at 4°C. Then, the supernatants were incubated with either Ni-NTA (Qiagen, 30210) or glutathione sepharose (GE Healthcare, 17-0756-01) beads at 4°C overnight. Subsequently, the beads were washed three to five times with lysis buffer (His_6_ precipitates) or PBS (GST precipitates). His_6_-tagged protein was released with His_6_ elution buffer (300 mM NaCl, 250 mM imidazole, 50 mM NaH_2_PO_4_ [pH8.0]). Purified proteins were checked for integrity and purity by Coomassie Brilliant Blue staining of polyacrylamide gel-fractionated samples. His_6_-tagged STAT3 was incubated with (bead-bound) GST or GST-vIRF-1 at 4°C overnight. Following incubation, the beads were washed 5 times by repeated precipitation and resuspension in ice-cold TBS. The beads were boiled in SDS-PAGE loading buffer (10 min.), prior to SDS-PAGE fractionation and immunoblotting of released proteins.

For endogenous vIRF-1 immunoprecipitation, TRExBCBL1-RTA cells were pelleted and washed in PBS prior to disruption in lysis buffer (50 mM Tris-HCl [pH 7.5], 150 mM NaCl, 5 mM EDTA, 0.2% NP40) containing protease inhibitor cocktail (Sigma, P8340) and sonication. After centrifugation at 15,000 × g for 15 min at 4°C, samples of the cleared lysates were incubated with either non-immune (control) rabbit antiserum or vIRF-1-specific rabbit antiserum for 1 h at 4°C. Protein A/G agarose beads (Santa Cruz, sc-2003) were then added, and incubation at 4°C continued overnight. The beads and associated proteins were washed three to five times with wash buffer (50 mM Tris-HCl [pH 7.5], 150 mM NaCl and 0.2% NP40) and were then boiled in SDS-PAGE loading buffer (10 min.), prior to SDS-PAGE fractionation and immunoblotting of released proteins.

### Antibodies

Primary antibodies used for immunoblotting were: CBD (New England BioLabs, E8034S); Flag (Sigma, F1804); β-actin (Sigma, A5316); GAPDH (Invitrogen, TAB1001); vIRF-1 (rabbit polyclonal antiserum, provided by Dr. Gary Hayward); vIL-6 (rabbit polyclonal antiserum [70]); STAT1, STAT2, STAT3, c-Myc and GST from Santa Cruz Biotechnologies (sc-464, sc-1668, sc482, sc-764 and sc-138, respectively); pSTAT3, TYK2 and JAK1 from Cell Signaling Technology (9131, 14193, 3344); LANA (Advanced Biotechnologies, 13-210-100), p-TYK2 (Cell Signaling Technology, 68790; BD Biosciences, 558394). HRP-conjugated anti-rabbit-IgG secondary, detection antibody was obtained from Cell Signaling Technology (7074) and from Rockland (18-8816-33), and anti-mouse IgG detection antibody was from Cell Signaling Technology (7076).

### Luciferase reporter assays

For reporter assays using 293T cells, ~60%-confluent cultures in 12-well tissue culture plates were PEI-cotransfected with 0.05 μg 4xM67-pTATA-Luc or pTATA-Luc, 0.005 μg pRL-TK (Renilla luciferase normalization control), and 0.5 μg of vIRF-1, vIRF-1 mutant or control expression vector. After 6 hours, transfection medium was replaced with fresh DMEM (with 10% FBS) with or without supplementation with vIL-6 conditioned medium. After 24 h, cells were lysed with lysis buffer prior to luciferase assays. For HHV-8 infected iSLK cells, ~60%-confluent cells in 12-well tissue culture plates were cotransfected using lipofectamine 2000 with 0.05 μg 4xM67-pTATA-Luc and 0.005 μg pRL-TK. After 6 hours, transfection medium was replaced with fresh DMEM/10% FBS containing doxycycline (1.9 nM) and sodium butyrate (1 mM), and cells were lysed after 48 h. Cell lysates were analyzed for firefly and Renilla luciferase activity using commercial reagent (Promega, E2920) according to the manufacturer’s instructions. Relative firefly luciferase activities were calculated after normalization to Renilla luciferase activity, to account for any differences in transfection efficiencies between cultures.

### HHV-8 mutagenesis

Genetic manipulation of the HHV-8 genome was carried out using the BAC16 bacmid genome of HHV-8 [35], essentially as outlined previously [36,71]. The coinsertion of mutations and a kanamycin resistance (Kan^r^) cassette and subsequent removal of the latter to leave only the intended mutations were checked by PCR amplification using primers flanking vIRF-1 locus and subsequent sequencing. HHV-8 BAC16 containing a mutation in the initiator-ATG codon of the vIRF-1 ORF (BAC16.vIRF-1^ttg^), creating a vIRF-1-knockout virus, was described previously [30]. A genome containing a deletion within the vIRF-1 ORF to remove residues 198 to 222 (Δ9) of the protein was generated by homologous recombination between a PCR fragment containing the Kan^r^ expression cassette flanked by Δ9-flanking sequences, with a reiterated sequence element in each arm to allow subsequent removal of Kan^r^ via a second intramolecular recombination. Reversion to wild-type sequences to generate a control, repaired virus was done in the same manner, but using a PCR fragment containing the sequences removed in the Δ9 mutation to restore the full-length vIRF-1 ORF. The primers used for generation of the PCR fragments used for mutagenesis and repair of Δ9-region sequences were as follows: Δ9–5’ forward: 5’-TTTCTCAGGATGGACACCATTTTCT-3’; Δ9–5’ reverse: 5’- CCCTACTTATCGTCGTCATCCTGGCGTTACAATCTCG CCTGGCAAGC-3’; Kan^r^ forward: 5’-AGGATGACGACGATAAGTAGGG-3’; Kan^r^ reverse: 5’-CAACCAATTAACCAATTCTGATTAG-3’; Δ9–3’ reverse: 5’- AAATCGTGGACGGCT CCGGCCACGACTCCACATTCCACGCATTGTTCTTCCAACCAATTAACCAATTCTGATTAG -3’; vIRF-1 repair reverse: 5’- GGCGTTACAATCTCGCCTGGCAAGCACTGCCCCGG GGAAAAAAATCCCTCCAACCAATTAACCAATTCTGATTAG -3’. Primers Δ9–5’ forward and Δ9–5’ reverse (applied to vIRF-1Δ9-ORF- or vIRF-1-ORF-containg plasmid), Kan^r^ forward and Kan^r^ reverse (applied to pEP-kanS; Addgene, #41017), and Δ9–5’ forward and Δ9–3’ reverse or vIRF-1 repair reverse (applied to the Δ9–5’ and Kan^r^ PCR products) were used to generate and assemble overlapping PCR fragments to produce a contiguous PCR fragment for recombination with BAC16 in GS1783 bacterial cells. The Kan^r^ cassette was removed by secondary recombination between flanking homologous sequences after BAC16 linearization by I-SceI cleavage within the Kan^r^ cassette. BAC16 genomes expressing vIRF-1 deleted of residues 23–47 (Δ2) and 48–72 (Δ3) and repaired versions thereof were generated similarly (by Dr Hyunwoo Ju). Gross genome integrities were checked by SpeI restriction digestion and comparisons of restriction profiles to that of native BAC16 on 0.8% agarose gels (run at 12 V for ∼20 h). BAC16 genomes were transfected into iSLK cells by using Fugene 6 (Promega, E2691), and BAC16^+^ cells were selected by hygromycin (2.3 mM) treatment of cultures for 3 weeks. Virus was recovered from the cultures by treatment with doxycycline (1.9 nM) and sodium butyrate (1 mM), and virus-containing media were harvested after 5 days to provide virus stocks. Infectious virus titers were determined based on the percentage of GFP^+^ cells resulting from inoculations of iSLK cultures using a dilution series of the virus stocks. Equivalent titers of virus were used to infect fresh iSLK or iTIME cells to provide “normalized” cultures for phenotypic analyses; normalizations were verified by LANA immunoblotting and/or qPCR of latent BAC16 genomes.

## Supporting information

S1 Fig Testing of vIRF-1 Δ2, Δ3 and Δ9 mutations for effects on vIRF-1 interactions with STAT1, STAT2 and STAT3. (A) Coprecipitation assays involving immunoprecipitation (IP) of Flag-tagged vIRF-1, expressed in transfected 293T cells, and immunoblot-detected endogenous STAT2 and STAT3. Arrowheads indicate the position of STAT2. RFP was used as a negative control. (B) Similar assays incorporating vector-expressed STAT1 (CBD-tagged) to facilitate its detection.
(TIF)

S2 Fig RT-qPCR analyses of HHV-8 lytic transcripts derived from Dox/NaB-induced wild-type (WT), vIRF-1Δ9 (Δ9) and vIRF-1Δ9-repaired (Δ9^R^) virus-infected iSLK cells. Data are from triplicate cultures, with average, GAPDH mRNA-normalized values calculated relative to the average “WT lytic” values (set at 1); error bars represent standard deviations from the average values for each dataset. Lytic replication was induced by Dox/NaB treatment for 2 days, and a set of cultures was untreated (latent, control).
(TIF)

S3 Fig Restriction analyses of BAC16 recombinant genomes and LANA-verified viral loads in infected iSLK cells. (A) Restriction endonuclease (SpeI) analysis of the gross integrity of the vIRF-1Δ9-expressing and vIL-6-ablated genome (Δ9+v6^ttg^) relative to wild-type BAC16 (WT), BAC16.vIRF-1Δ9 (Δ9), and BAC16.vIL-6^ttg^ (v6^ttg^) [41] genomes. The white and grey arrowheads indicate fragments containing wild-type and Δ9-mutated vIRF-1 ORFs; the black arrowhead indicates the vIL-6 ORF-containing fragment. (B) Immunoblot verification of equivalent LANA expression in iSLK cultures infected with the same infectious doses of wild-type, vIRF-1Δ9, vIL-6^ttg^, and vIRF-1Δ9/vIL-6^ttg^ viruses. (C) SpeI restriction profiling of vIRF-1Δ2 (Δ2) and vIRF-1Δ3 (Δ3) BAC16 genomes, expressing STAT3-binding-refractory vIRF-1 proteins, and wild-type-reverted derivatives (Δ2^R^, Δ3^R^). Grey and white arrowheads indicate bands containing mutated and wild-type vIRF-1 ORFs, respectively. (D) LANA-immunoblot confirmation of equivalent viral loads in iSLK cultures infected with an equal infectious dose of each virus.
(TIF)

S4 Fig Testing for potential effects of vIRF-1-STAT3 interaction-promoting pep.1-72 on inhibition of vIL-6-activated STAT3 signaling by vIRF-1. A STAT3 reporter (4xM67-Luc) was transfected into 293T cells along with empty vector (-vIL-6) or vIL-6 expression plasmid (+vIL-6) and either RFP (negative control) or vIRF-1 expression plasmids; pep.1-72 vector was added to one set of triplicate cultures expressing vIRF-1. Luciferase activities from transfected cultures were averaged for each set of biological replicates and quantified relative to RFP-expressing cells without vIL-6 coexpression (Rfp, -vIL-6); standard deviations from average values are shown. Immunoblots below the chart verify appropriate protein/peptide expression (combined replicates).
(TIF)

S5 Fig Analysis of expression of HHV-8 lytic proteins, along with latent/lytic LANA, in response to pep.1-72. HHV-8-infected iSLK cells were transduced with a lentiviral vector expressing pep.1-72 or RFP (control) and the parallel cultures were then treated with Dox/NaB for 2 days prior to generation and immunoblot analysis of cells extracts. Two experiments (expt. 1, expt. 2) were performed, showing substantially increased expression of vIRF-1, but not other viral proteins, in cells expressing pep.1-72 (1–72).
(TIF)

S6 Fig Effects of pep.1-72 and STAT3 depletion on vIRF-1 expression in iTIME and iBCBL-1 cells. (A) Lentiviral vector-transduced iTIME cells expressing RFP (control) or pep.1-72 and latently infected with HHV-8 were treated with Dox/NaB to induce lytic replication and media were harvested after 4 days for qPCR determination of relative virus yields. Data are expressed as average values from biological triplicates; standard deviations and student t-test *P* value are shown. Parallel cultures were harvested 2 days after lytic induction for immunoblot analysis of vIRF-1 expression relative to other viral proteins. (B) An analogous experiment was carried out in iBCBL-1 cells treated for 2 days with Dox to induce lytic replication. Lysates of cells harvested 1 day after lytic induction were immunoblotted for analysis of vIRF-1 expression. The dotted line indicates lane deletion. (C) Expression of vIRF-1 and other viral proteins in response to shRNA-mediated depletion of STAT3 in HHV-8^+^ iTIME cells, treated with Dox/NaB for 2 days prior to cell harvest. Lentiviral vectors were used to express STAT3 mRNA-directed or non-silencing (ns) control shRNAs (sh). Two experiments (expt. 1, expt. 2) were performed. (D) Equivalent analyses were carried out in iBCBL-1 cells, harvested 1 day after lytic induction with Dox.
(TIF)

S1 Data Compiled primary and processed data underlying charts presented in the manuscript. (XLSX)
