# Supplementary figures and images for: Direct and biologically significant interactions of human herpesvirus 8 interferon regulatory factor 1 with STAT3 and Janus kinase TYK2
Source: PLoS Pathog. 2023 Nov 20;19(11):e1011806. doi: 10.1371/journal.ppat.1011806 (PMC10695398; doi:10.1371/journal.ppat.1011806)

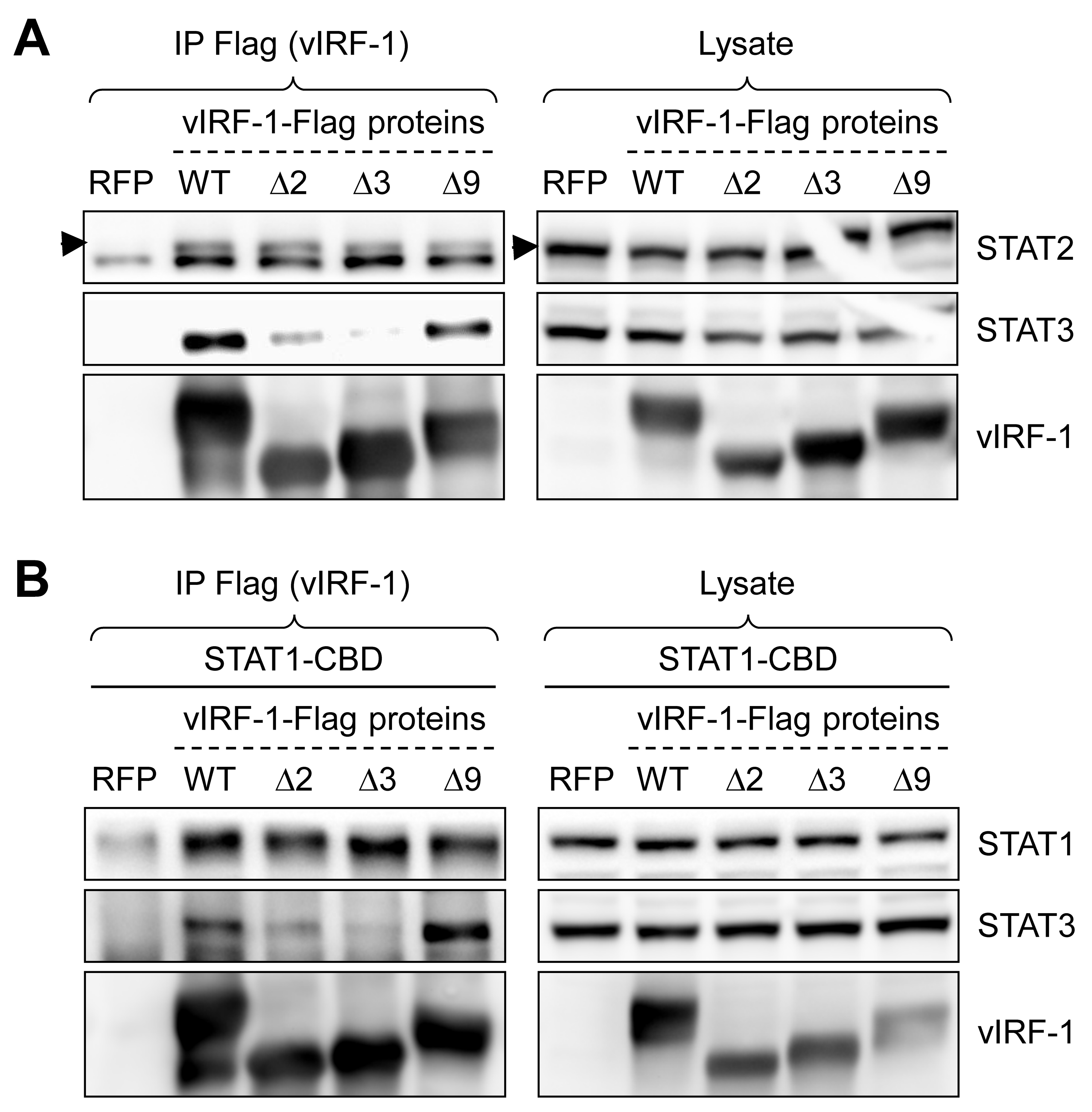

Supplement: S1 Fig — (A) Coprecipitation assays involving immunoprecipitation (IP) of Flag-tagged vIRF-1, expressed in transfected 293T cells, and immunoblot-detected endogenous STAT2 and STAT3. Arrowheads indicate the position of STAT2. RFP was used as a negative control. (B) Similar assays incorporating vector-expressed STAT1 (CBD-tagged) to facilitate its detection. (TIF) [file ppat.1011806.s001.tif]

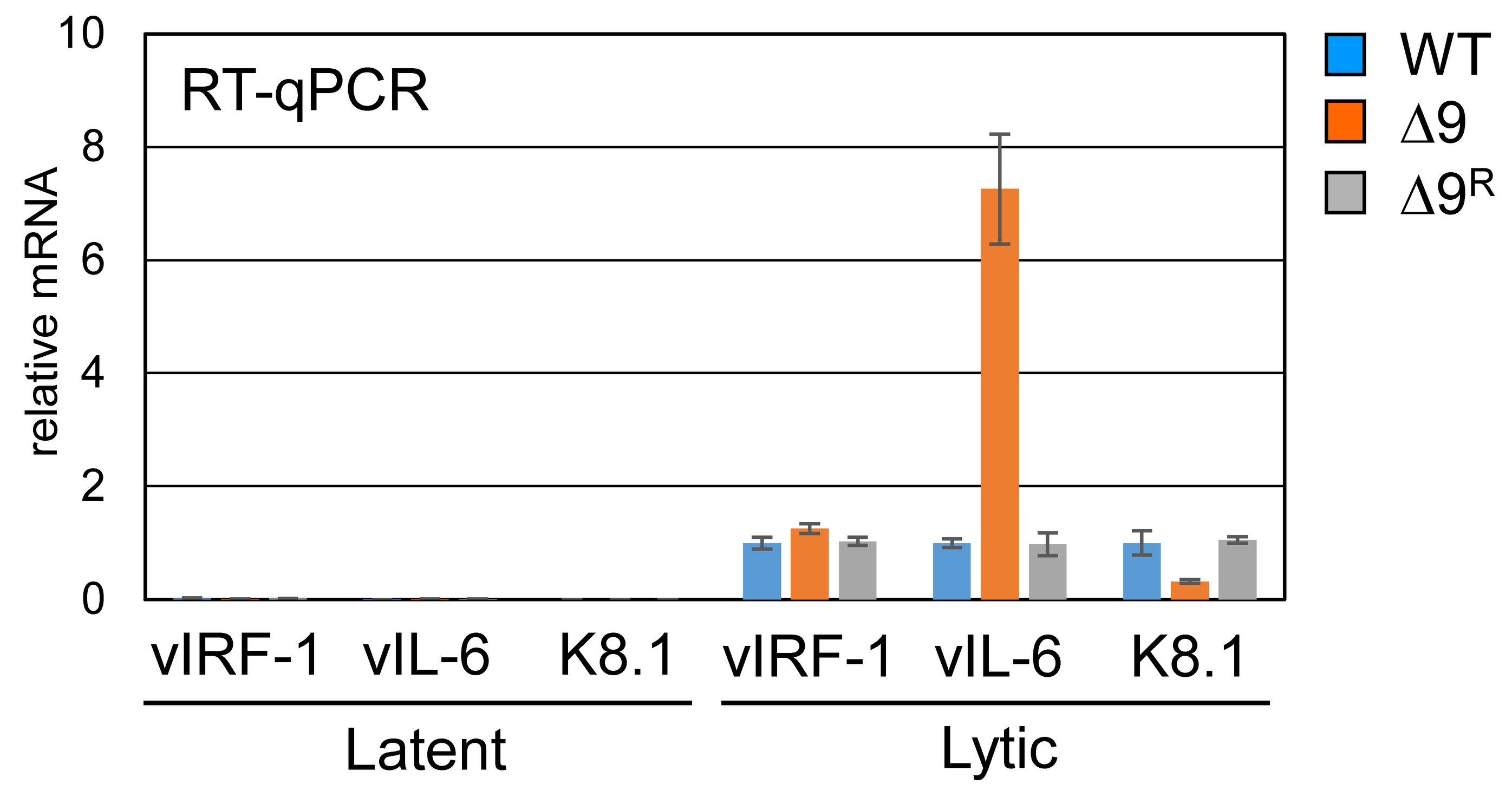

Supplement: S2 Fig — Data are from triplicate cultures, with average, GAPDH mRNA-normalized values calculated relative to the average “WT lytic” values (set at 1); error bars represent standard deviations from the average values for each dataset. Lytic replication was induced by Dox/NaB treatment for 2 days, and a set of cultures was untreated (latent, control). (TIF) [file ppat.1011806.s002.tif]

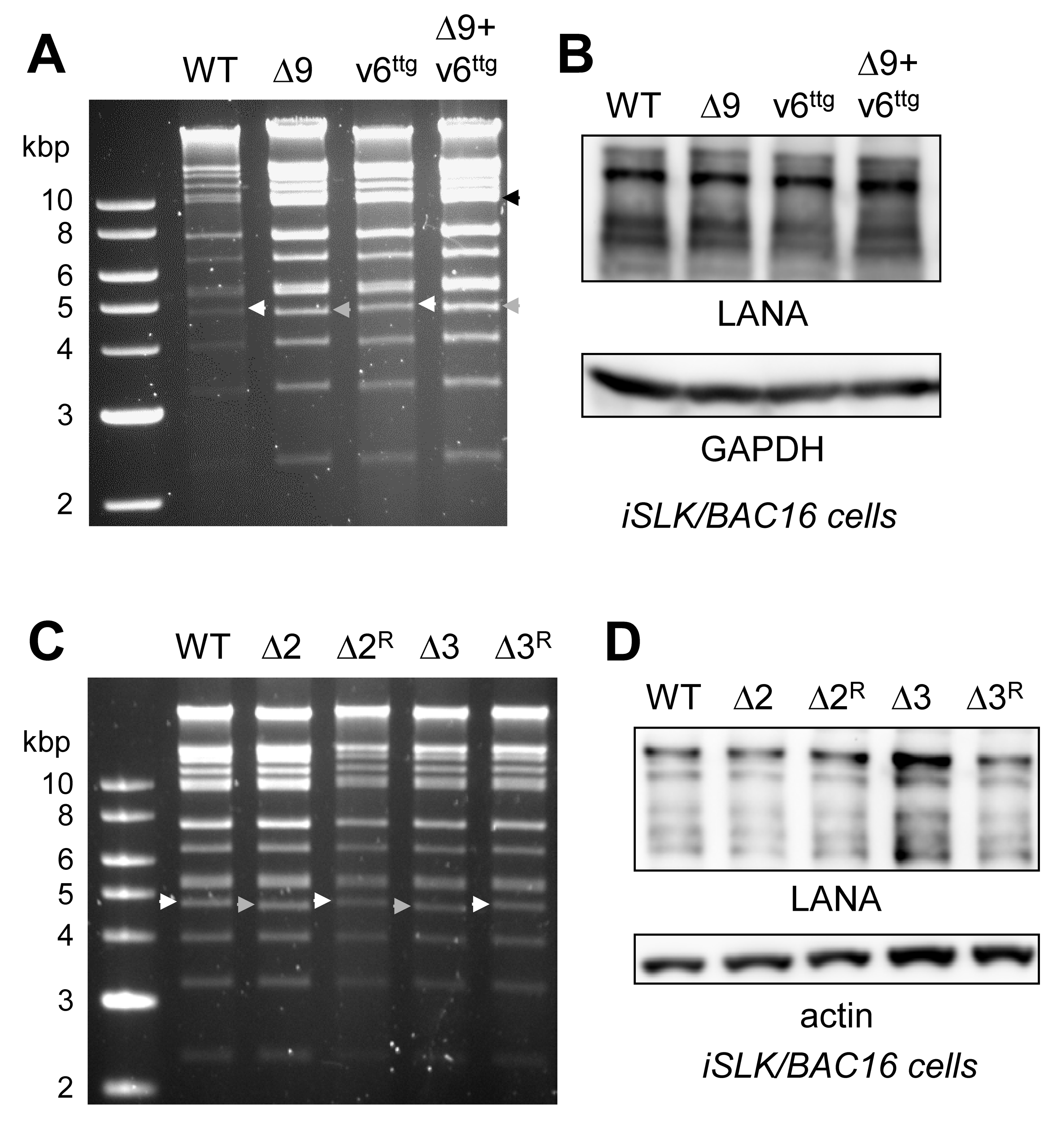

Supplement: S3 Fig — (A) Restriction endonuclease (SpeI) analysis of the gross integrity of the vIRF-1Δ9-expressing and vIL-6-ablated genome (Δ9+v6ttg) relative to wild-type BAC16 (WT), BAC16.vIRF-1Δ9 (Δ9), and BAC16.vIL-6ttg (v6ttg) [41] genomes. The white and grey arrowheads indicate fragments containing wild-type and Δ9-mutated vIRF-1 ORFs; the black arrowhead indicates the vIL-6 ORF-containing fragment. (B) Immunoblot verification of equivalent LANA expression in iSLK cultures infected with the same infectious doses of wild-type, vIRF-1Δ9, vIL-6ttg, and vIRF-1Δ9/vIL-6ttg viruses. (C) SpeI restriction profiling of vIRF-1Δ2 (Δ2) and vIRF-1Δ3 (Δ3) BAC16 genomes, expressing STAT3-binding-refractory vIRF-1 proteins, and wild-type-reverted derivatives (Δ2R, Δ3R). Grey and white arrowheads indicate bands containing mutated and wild-type vIRF-1 ORFs, respectively. (D) LANA-immunoblot confirmation of equivalent viral loads in iSLK cultures infected with an equal infectious dose of each virus. (TIF) [file ppat.1011806.s003.tif]

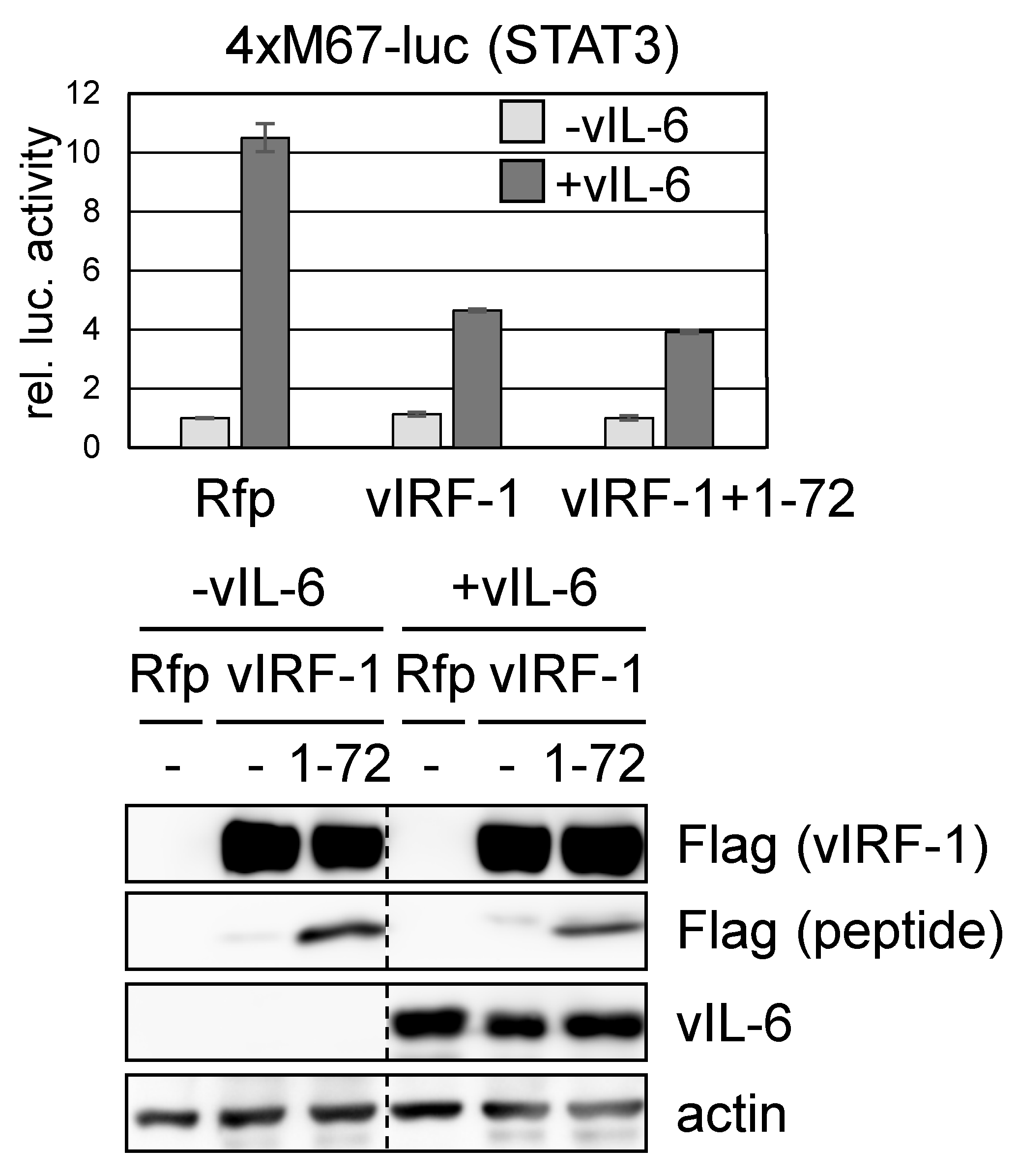

Supplement: S4 Fig — A STAT3 reporter (4xM67-Luc) was transfected into 293T cells along with empty vector (-vIL-6) or vIL-6 expression plasmid (+vIL-6) and either RFP (negative control) or vIRF-1 expression plasmids; pep.1-72 vector was added to one set of triplicate cultures expressing vIRF-1. Luciferase activities from transfected cultures were averaged for each set of biological replicates and quantified relative to RFP-expressing cells without vIL-6 coexpression (Rfp, -vIL-6); standard deviations from average values are shown. Immunoblots below the chart verify appropriate protein/peptide expression (combined replicates). (TIF) [file ppat.1011806.s004.tif]

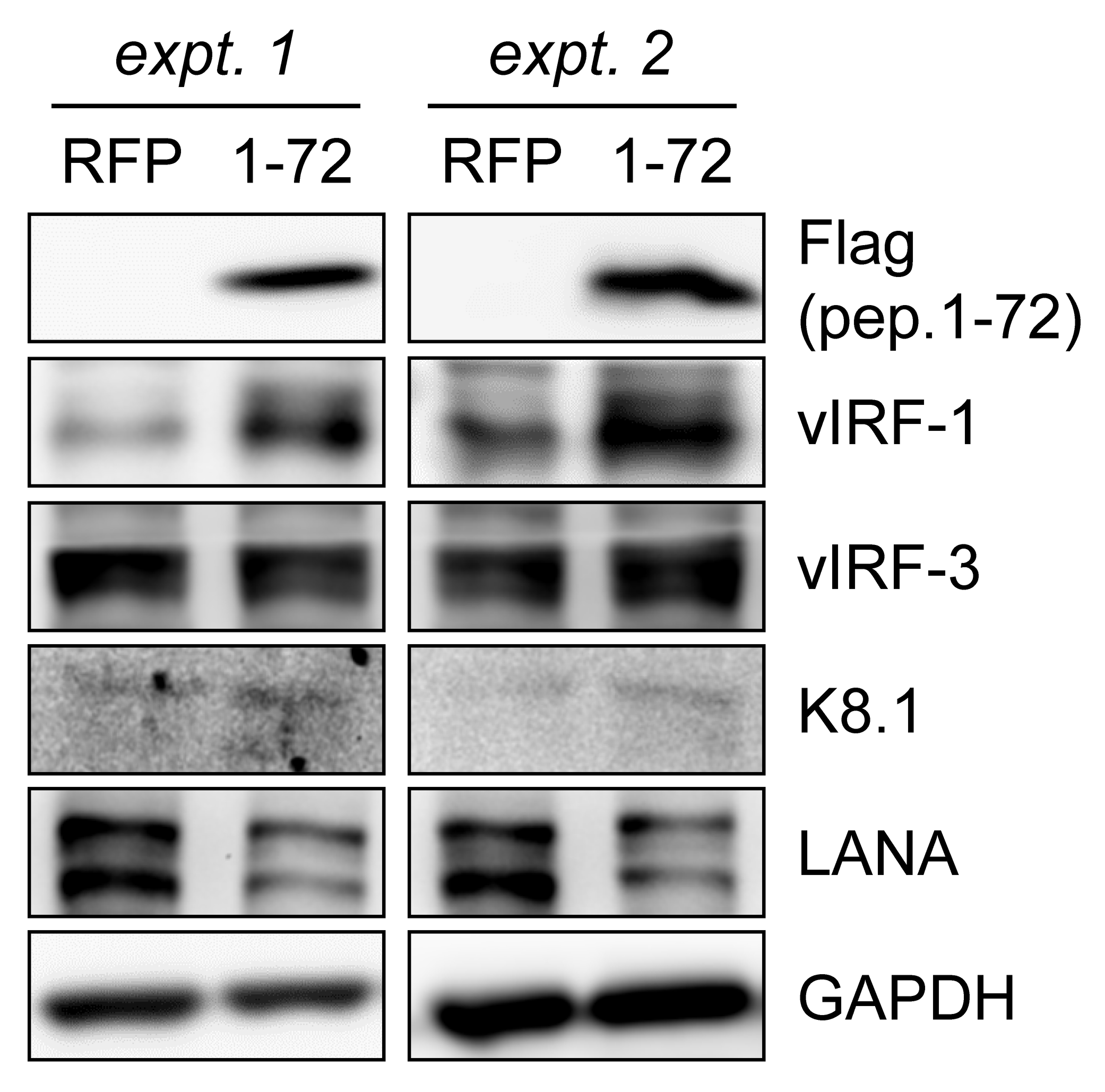

Supplement: S5 Fig — HHV-8-infected iSLK cells were transduced with a lentiviral vector expressing pep.1-72 or RFP (control) and the parallel cultures were then treated with Dox/NaB for 2 days prior to generation and immunoblot analysis of cells extracts. Two experiments (expt. 1, expt. 2) were performed, showing substantially increased expression of vIRF-1, but not other viral proteins, in cells expressing pep.1-72 (1–72). (TIF) [file ppat.1011806.s005.tif]

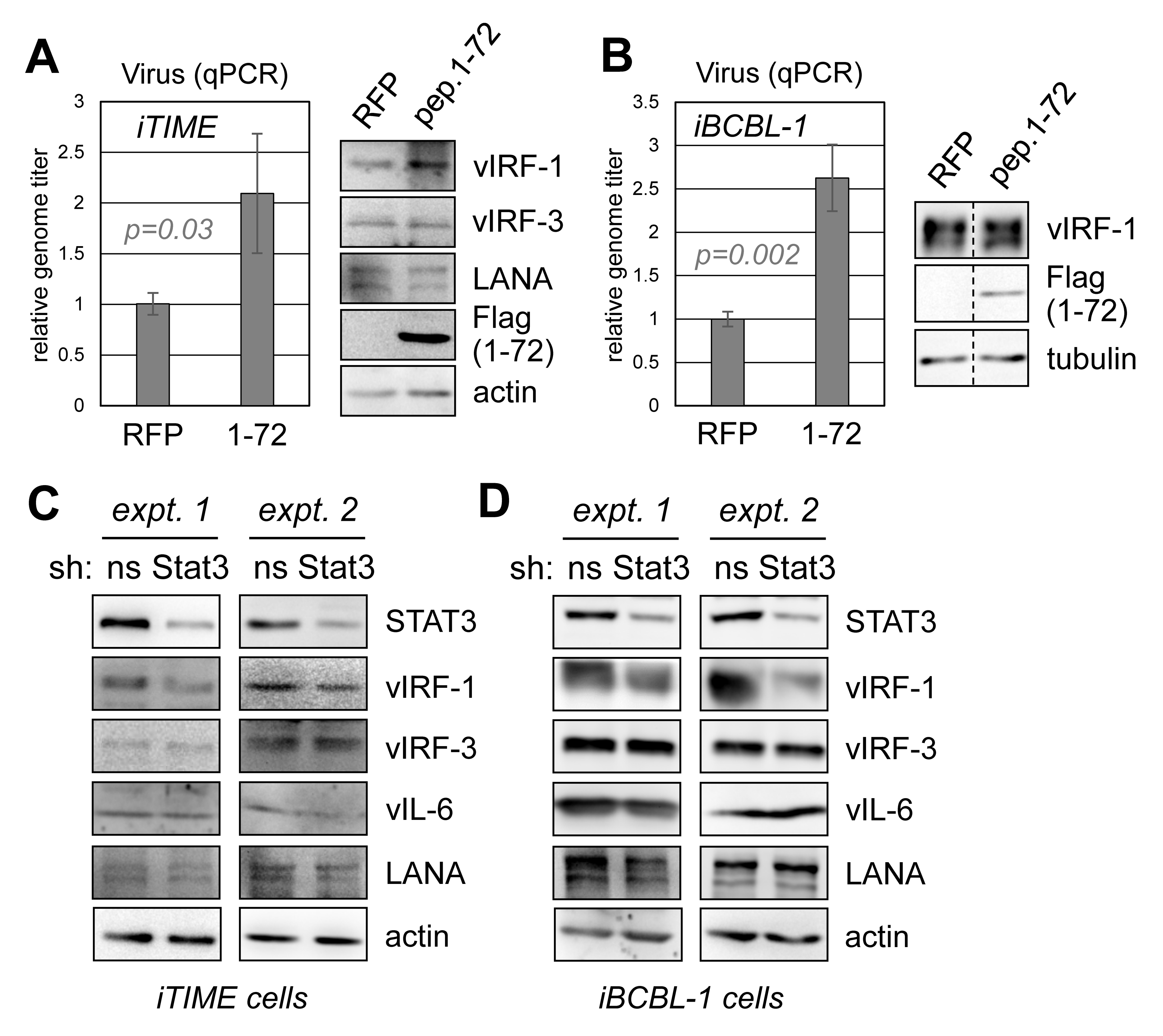

Supplement: S6 Fig — (A) Lentiviral vector-transduced iTIME cells expressing RFP (control) or pep.1-72 and latently infected with HHV-8 were treated with Dox/NaB to induce lytic replication and media were harvested after 4 days for qPCR determination of relative virus yields. Data are expressed as average values from biological triplicates; standard deviations and student t-test P value are shown. Parallel cultures were harvested 2 days after lytic induction for immunoblot analysis of vIRF-1 expression relative to other viral proteins. (B) An analogous experiment was carried out in iBCBL-1 cells treated for 2 days with Dox to induce lytic replication. Lysates of cells harvested 1 day after lytic induction were immunoblotted for analysis of vIRF-1 expression. The dotted line indicates lane deletion. (C) Expression of vIRF-1 and other viral proteins in response to shRNA-mediated depletion of STAT3 in HHV-8+ iTIME cells, treated with Dox/NaB for 2 days prior to cell harvest. Lentiviral vectors were used to express STAT3 mRNA-directed or non-silencing (ns) control shRNAs (sh). Two experiments (expt. 1, expt. 2) were performed. (D) Equivalent analyses were carried out in iBCBL-1 cells, harvested 1 day after lytic induction with Dox. (TIF) [file ppat.1011806.s006.tif]
